# *Acinetobacter baumannii* OmpA hinders host autophagy via the CaMKK2-reliant AMPK-pathway

**DOI:** 10.1128/mbio.03369-24

**Published:** 2025-02-25

**Authors:** Kyungho Woo, Dong Ho Kim, Ho-Sung Park, Man Hwan Oh, Je Chul Lee, Chul Hee Choi

**Affiliations:** 1 Department of Microbiology, School of Medicine, Chungnam National University 90159 , Daejeon, South Korea; 2 Translational Immunology Institute, School of Medicine, Chungnam National University, Daejeon, South Korea; 3 Department of Medical Science, School of Medicine, Chungnam National University 90159 , Daejeon, South Korea; 4 System Network Inflammation Control Research Center, School of Medicine, Chungnam National University, Daejeon, South Korea; 5 Department of Microbiology, College of Science and Technology, Dankook University, Cheonan, South Korea; 6 Department of Microbiology, School of Medicine, Kyungpook National University, Daegu, South Korea; The Ohio State University College of Nursing, Columbus, Ohio, USA; The Research Institute at Nationwide Children's Hospital, Columbus, Ohio, USA

**Keywords:** *Acinetobacter baumannii*, autophagy, CaMKK2, outer membrane protein A

## Abstract

Outer membrane protein A (OmpA) plays a vital role in the interactions between *Acinetobacter baumannii* and host cells. Autophagy is a defense mechanism that hinders the intracellular replication of bacteria, thereby safeguarding cells against microbial infections. While it has been observed that *A. baumannii* triggers cellular autophagy, the precise role of its virulence protein OmpA in this process remains uncertain. In this study, we investigated the effects of *A. baumannii* OmpA (AbOmpA) on autophagy and explored the underlying molecular mechanisms. We found that AbOmpA exerted its autophagy-suppressive effect through inhibition of CaMKK2 phosphorylation. Compared to the wild-type strain, the *ompA*-deletion mutant strain displayed considerably enhanced autophagy induction, *via* the AMPK-ULK1 pathway. AbOmpA hindered starvation-induced autophagy, while *A. baumannii*-Omp33 (AbOmp33) and *Escherichia coli*-OmpA (EcOmpA) did not. Importantly, we confirmed that exogenous AbOmpA suppressed autophagy through the CaMKK2-AMPK-ULK1 pathway during *A. baumannii* infection. These findings reveal a novel mechanism for AbOmpA-mediated autophagy evasion, providing new insights into the pathogenesis of *A. baumannii* infection.

**IMPORTANCE:**

*Acinetobacter baumannii* is a significant clinical pathogen notorious for causing infections in hospitals. Its outer membrane protein A acts as a virulence factor and helps the bacteria evade host defenses. Autophagy is a defense mechanism that hinders the intracellular replication of bacteria. While it has been observed that *A. baumannii* triggers cellular autophagy, the precise role of its AbOmpA in this process remains uncertain. Our studies demonstrate the AbOmpA of *A. baumannii* inhibits the cellular defense process, autophagy, through the CaMKK2-AMPK-ULK1 signaling cascade, thereby enhancing bacterial survival. This insight into how AbOmpA bypasses autophagy sheds light on *A. baumannii* infection's novel virulence strategy and suggests possible treatments.

## INTRODUCTION

*Acinetobacter baumannii* is a clinically important pathogen with ubiquitous environmental distribution and high hospital infectivity potential (1). Its ability to cause infections at various anatomical sites, particularly in the respiratory and urinary tracts, renders it a major challenge in surgical wards and intensive care units (2). The emergence of multidrug-resistant *A. baumannii* strains underscores the need for alternative approaches to traditional antibiotic treatment, for the management of antibiotic-resistant infections (3, 4). Outer membrane protein A (AbOmpA) expression in *A. baumannii* is linked to nosocomial pneumonia and bacteremia-associated mortality; in addition, it serves as a critical diagnostic marker for antibiotic resistance (5). Since it is a virulence factor in evading host defense systems during infection, the outer membrane protein (OMP) has attracted attention as a target for novel therapeutic strategies (5, 6).

OMPs are β-barrel structures of integral membrane proteins with structural features that contribute to their high stability and allow them to withstand harsh and variable environments (7). Among the different OMPs, the virulence factor AbOmpA from *A. baumannii* has been extensively studied and plays a crucial role in diverse biological processes, such as regulating adhesion, aggression, internalization, immune response, and biofilm-formation. Specifically, AbOmpA is indispensable for *A. baumannii* attachment to and invasion of epithelial cells, and the interaction between *A. baumannii* and host cells is initiated by the binding of AbOmpA to fibronectin (5). AbOmpA is also responsible for the induction of apoptosis and generation of reactive oxygen species (8).

Autophagy is a cellular process that involves the degradation and recycling of proteins into nutrients and removal of damaged cellular organelles (9). The regulation of this process is mediated by microtubule-associated protein 1 light chain 3 (LC3) and ATG, with the formation of an LC3–phosphatidylethanolamine conjugate (LC3-II) on the autophagic membrane, which contributes to subsequent autophagosome formation (10). The cargo is engulfed by the layered isolation membranes of the organelle to form double-membrane autophagosomes, which ultimately fuse with lysosomes to form autolysosomes, leading to lysosomal degradation (11). The autophagy initiator, Unc-51-like kinase 12 (ULK1), forms a complex that induces autophagy and is activated by phosphorylation induced by upstream regulators, such as the mammalian target of rapamycin (mTOR) and AMP-activated protein kinase (AMPK) pathway (12). Liver kinase B1 (LKB1) acts as an upstream kinase that enhances AMPK activity by means of phosphorylation, while calmodulin-dependent protein kinase kinase 2 (CaMKK2) activates AMPK in the presence of high cytosolic calcium (13).

Previous studies have shown that *A. baumannii* triggers autophagy through the mTOR signaling pathway in the *in vivo* Sprague–Dawley (SD) mouse model and the AMPK/extracellular signal-related kinase (ERK)/mTOR signaling pathway in epithelial cells (14, 15). Autophagy is also induced by AbOmpA and AbOmp33 (16, 17). However, several pathogenic bacteria have developed mechanisms to evade autophagic flux and host defenses. For instance, *Escherichia coli* O157:H7 hinders host autophagy and persists in intestinal epithelial cells (18). Other bacteria, such as *Mycobacterium tuberculosis* ESAT-6 secretion system (ESX1), *Shigella flexneri* virulence factor VirG, and *Listeria monocytogenes* toxin listeriolysin, block bacterial recruitment to the phagophore (19). Conversely, *A. phagocytophilum*, *Yersinia pseudotuberculosis*, *Coxiella burnetii*, and *Francisella tularensis* induce the formation of autophagic vesicles or accumulation of autophagosomes as a nutrient source for microbial growth (20). Increased autophagy through anti-inflammatory effects may unintentionally help bacteria survive (21).

Although most autophagy studies have focused on intracellular bacteria, extracellular bacterial clearance by autophagy also occurs in a similar manner. Autophagy is induced by the stimulation of TLRs or PAMPs by extracellular bacteria (22, 23). Some extracellular bacteria—*Vibrio cholerae*, extracellular enteropathogenic *Escherichia coli* (EPEC), *Staphylococcus aureus*, and *Pseudomonas aeruginosa* can inhibit host cell autophagy by their toxins (24–26). However, the interaction between extracellular bacteria and autophagy is still poorly understood, and in the case of *A. baumannii*, no clear toxin has been identified. The pathogenesis of *A. baumannii* is caused by a combination of factors (27). Moreover, while it has been reported that *A. baumannii* activates autophagy, the *A. baumannii*-mediated autophagy process is incompletely understood (14, 15, 17), and the molecular mechanisms underlying the inhibition and evasion of autophagy by *A. baumannii* remain largely unclear. In this study, we examined the impact of *A. baumannii* and AbOmpA on cellular autophagy and investigated the molecular mechanisms underlying autophagy inhibition.

## RESULTS

### *Acinetobacter baumannii* induces phosphorylation of the AMPK-ULK pathway in RAW 264.7 cells

**Fig 1:**
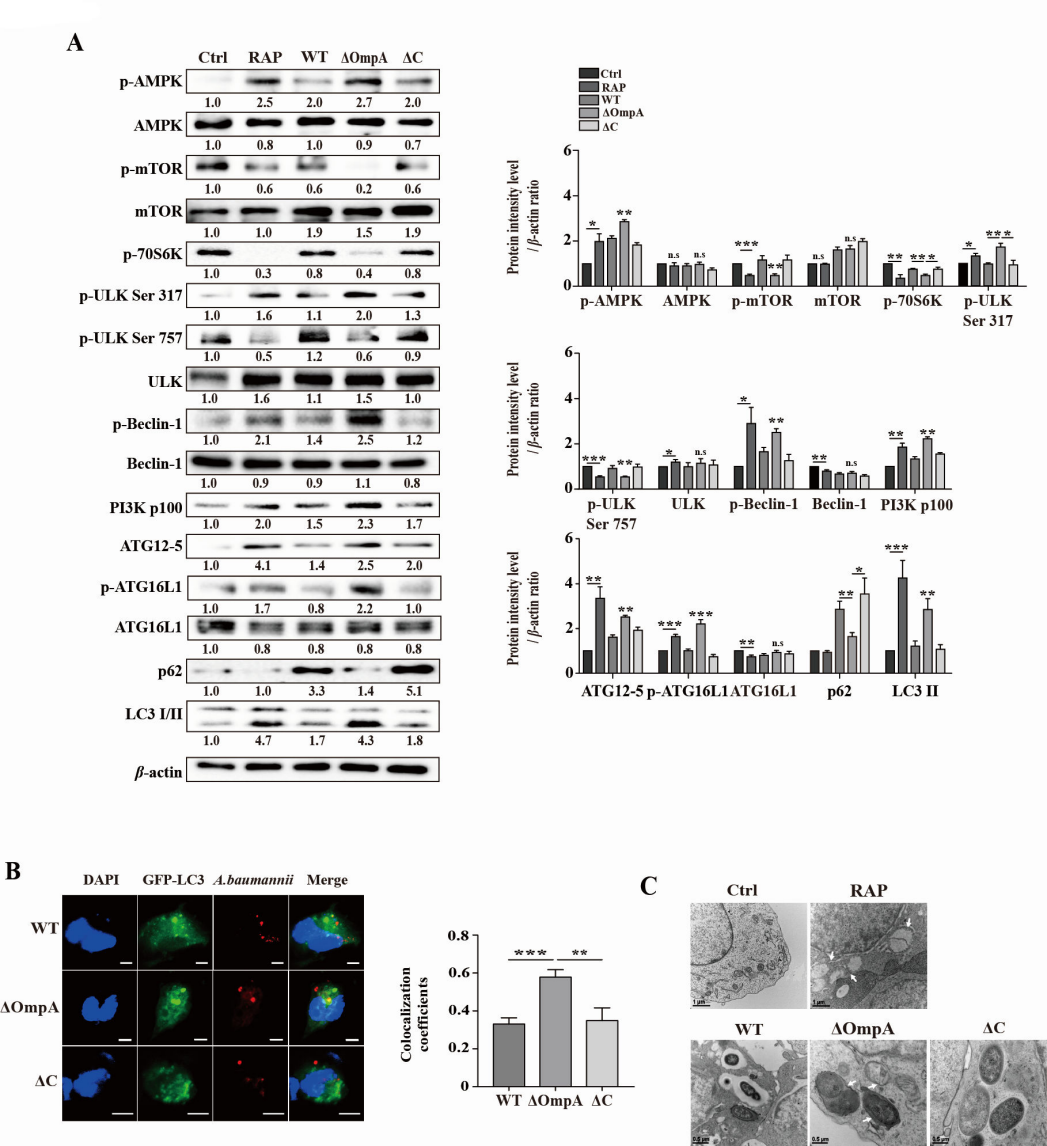
*Acinetobacter baumannii* induces autophagy through the AMPK/mTOR/ULK pathway in RAW 264.7 cells. RAW 264.7 cells were infected with *A. baumannii* ATCC 17978 wild-type (WT), *ompA*-deletion mutant (ΔOmpA), and *ompA*-complemented (ΔC) strains at a multiplicity of infection (MOI) of 100 for 6 hours. (**A**) The samples obtained from the infected cells were immunoblotted for target proteins. Target protein levels were normalized to those of *β*-actin and presented as mean ± SEM of three independent experiments. ***P < 0.001, **P < 0.01, and *P < 0.05 *versus* WT and ΔC. (**B**) The GFP-LC3-transfected cells were fixed, permeabilized, and incubated with anti-AbOmpA antibodies, following which they were visualized by means of confocal microscopy. Pearson’s correlation coefficient was calculated using JACoP (*n* = 5). Scale bar: 5 µm. Data are presented as the mean ± SEM. ***P < 0.001; **P < 0.01. (**C**) The infected cells were subjected to transmission electron microscopy after treatment with rapamycin (6 µM) for 6 hours. Scale bar: 0.5–1 µm. White arrowheads indicate double-membrane. MOI, multiplicity of infection. RAP, rapamycin. Ctrl, negative control.

When AMPK is activated in response to stress, it promotes autophagy by inhibiting the phosphorylation of mTORC1 and ULK Ser757. AMPK-mediated phosphorylation of ULK1 at Ser317 is induced, triggering autophagy initiation (28, 29). Previous studies have reported that *A. baumannii* infection promotes the activation of AMPK and Beclin-1, both of which are involved in autophagy (15). However, to compare the regulation and induction of autophagy in *A. baumannii* the wild-type (WT) and the AbOmpA-mutant strain infection, we investigated the AMPK-ULK pathway. The AbOmpA-mutant strain-infected cell displayed higher levels of AMPK and ULK Ser317 phosphorylation than the WT and complemented strain-infected cells (Fig. 1A). In contrast, mTORC1 activation inhibits autophagy by phosphorylating ULK1 at Ser757 and binding to the ULK1 complex. Rapamycin increases autophagy by inhibiting mTOR (30). Rapamycin treatment inhibited the activation of mTOR, 70S6K, and ULK1 Ser 757. Similarly, inhibition of mTOR, 70S6K, and ULK1 Ser 757 was observed in cells infected with the AbOmpA-mutant strain, with reduction than in those infected with the WT and complemented strains (Fig. 1A). For phagophore membrane nucleation, the Unc-51-like kinase 1 (ULK1)–ATG13–FIP200 (FAK family kinase-interacting protein of 200 kDa) complex is activated, and then the PI3K-III complex is formed by Beclin-1 (24). Autophagosome elongation is promoted by microtubule-associated protein 1 light chain 3 (LC3) and the ATG5–ATG12 conjugation system (31). To investigate the overall autophagy initiation process during *A. baumannii* infection, we examined ATG12–ATG5 conjugation, PI3K-III, and Beclin-1 expression. Cells infected with the AbOmpA mutant strain showed increased phosphorylation of Beclin-1 and ATG16L1, and ATG12-5 conjugation levels were higher than in WT and complemented strain-infected cells (Fig. 1A). The level of LC3-II was higher in AbOmpA-mutant strain-infected cells, while P62 expression was lower compared to that of WT and complemented strain-infected cells (Fig. 1A). Thus, autophagy induction was significantly higher in the AbOmpA-mutant strain-infected cells than in the wild-type strain-infected cells. After 6 hours of infection, the AbOmpA-mutant strain exhibited clear colocalization of GFP-LC3, whereas the WT and complemented strains showed only slight colocalization (Fig. 1B). Additionally, intracellular *A. baumannii* (WT and complemented strains) were freely observed in the cytosol and single-membrane phagosomes, whereas the AbOmpA-mutant strain exhibited distinct double-membrane structures (Fig. 1C). Taken together, these results suggested that AbOmpA is involved in restricting autophagy during the early stages of infection.

### AbOmpA inhibits autophagic vacuoles in RAW 264.7 cells

**Fig 2:**
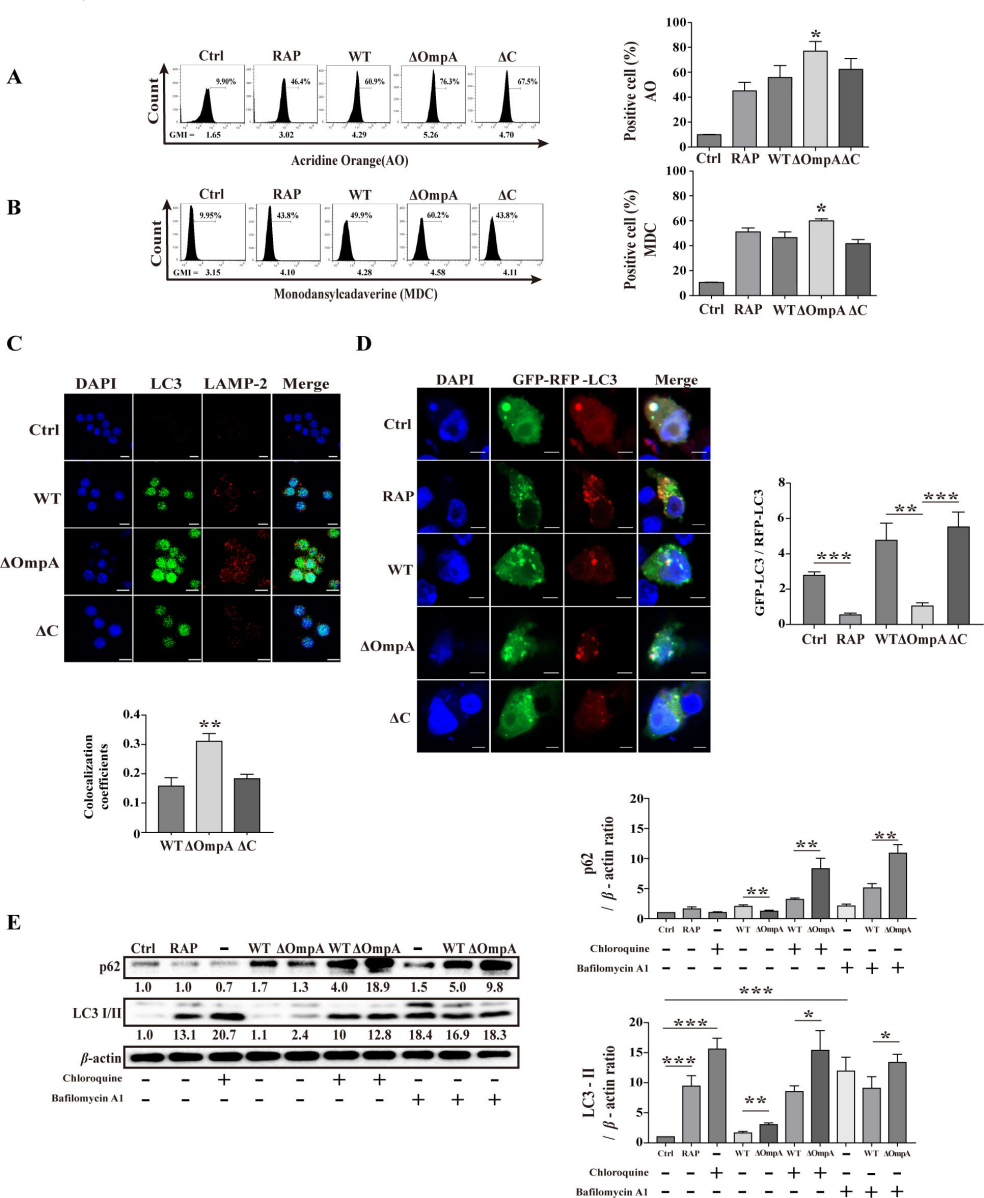
Analysis of the autophagic vacuoles in *Acinetobacter baumannii*. (**A**) RAW 264.7 cells were infected with WT, ΔOmpA, and ΔC, at an MOI of 100, for 6 hours. The cells were stained with AO and then subjected to flow cytometry analysis. (**B**) Cells were stained with monodansylcadaverine. Data are presented as mean ± SEM of three independent experiments. *P < 0.05 *versus* WT. (**C**) Representative confocal images of LC3 and LAMP2, after infection for 6 hours. Pearson’s correlation coefficient were calculated using JACoP (*n* ≥ 5). Scale bar: 10 µm. (**D**) Representative confocal images of transfected cells with GFP-LC3-RFP-LC3ΔG, after infection for 6 hours. GFP/RFP intensity ratios were calculated (*n* ≥ 5). Scale bar: 5 µm. Data are presented as the mean ± SEM. ***P < 0.001; **P < 0.01. (**E**) RAW 264.7 cells were infected with WT and ΔOmpA, at an MOI of 100, for 6 hours, and then treated with chloroquine (20 µM) for 2 hours and bafilomycin A1 for 4 hours, following which the cells were harvested to obtain samples for Western blot analysis. LC3-II and p62 levels were normalized to those of *β*-actin and presented as mean ± SEM of three independent experiments. ***P < 0.001, **P < 0.01, and *P < 0.05. MOI, multiplicity of infection; AO, acridine orange; RAP, rapamycin; WT, *A. baumannii* ATCC 17978 wild-type; ΔOmpA, *A. baumannii* ATCC 17978 *ompA*-deletion mutant strain; ΔC, Ctrl, negative control.

Autophagy breaks down invading bacteria through the fusion of acidic lysosomes with autophagosomes (32). To confirm the fusion of autophagosomes and lysosomes in each *A. baumannii*-infected RAW 264.7 cell, we assessed them using flow cytometry and confocal microscopy. Monodansylcadaverine was used to stain lipid-containing vacuoles such as autophagosomes and acridine orange was used to stain acidic vesicular organelles. While all *A. baumannii*-infected cells exhibited an increased number of acidic vacuoles in the cytoplasm, the AbOmpA mutant strain showed a 20% higher increase in acidic vacuoles than the WT strain (Fig. 2A). Additionally, there was a 10% difference in the number of lipid-containing vacuoles between the two strains (Fig. 2B). When we visualized lysosomal-associated membrane protein 2 (LAMP2; a marker of the acidic compartment of autolysosomes), the AbOmpA-mutant strain exhibited remarkably increased LAMP2-LC3B colocalization, whereas the WT and complemented strains exhibited only slight LAMP2-LC3B colocalization (Fig. 2C). For further measurements of autophagy flux, we transfected the GFP-LC3-RFP-LC3ΔG probe into RAW 264.7 cells and measured the GFP/RFP ratio to monitor the autophagic flux. The AbOmpA-mutant strain showed increased RFP-LC3 and GFP-RFP colocalization in RAW 264.7 cells (Fig. 2D). Additionally, the AbOmpA-mutant strain had lower GFP/RFP ratios compared to the wild-type (WT) and complemented strain-infected cells (Fig. 2D). We also performed experiments to analyze the differential accumulation of autophagosomes and LC3 lipidation following co-treatment with the autophagic flux-blocking agent chloroquine and bafilomycin A1. LC3-II and p62 accumulation was significantly higher in the AbOmpA-mutant strain than in the WT strain (Fig. 2E). These results indicated that the AbOmpA-mutant strain strongly induces autophagy and suggested that AbOmpA may have a suppressive effect on the autophagosome–lysosome fusion.

### Exogenous AbOmpA blocks the AMPK pathway

**Fig 3:**
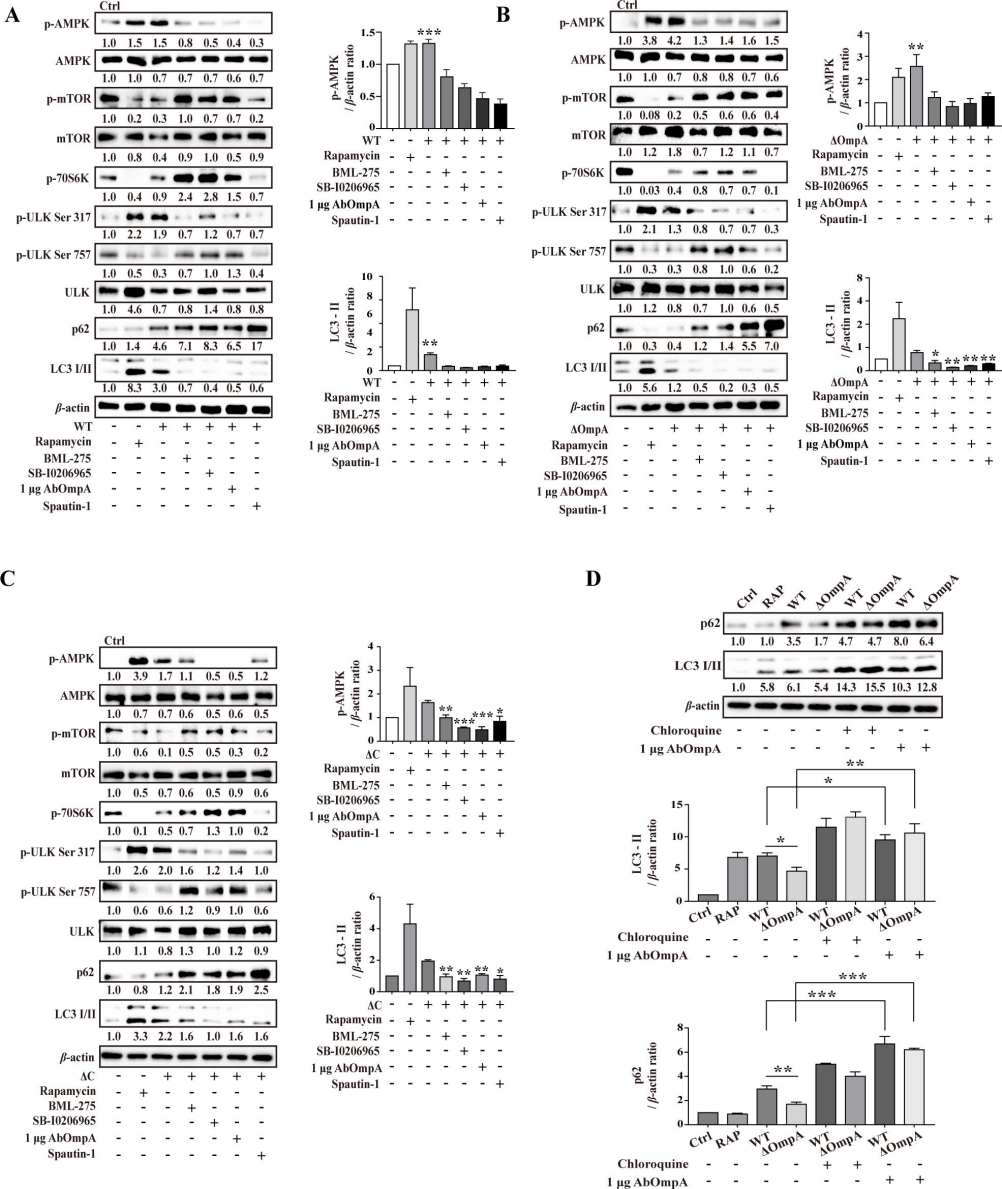
Exogenous AbOmpA inhibits the autophagy initiation mechanism. RAW 264.7 cells were infected with (**A**) WT, (**B**) ΔOmpA, and (**C**) ΔC, at an MOI of 100, for 6 hours. The cells were then simultaneously treated with BML-275 (AMPK inhibitor), SB-I0206965 (ULK inhibitor), spautin-1 (PI3 kinase class III complex inhibitor), rapamycin, and/or exogenous AbOmpA. All signal molecules were analyzed using Western blot. Target protein levels were normalized to those of *β*-actin and presented as mean ± SEM of three independent experiments. ***P < 0.001, **P < 0.01, and *P < 0.05 *versus* each of infection condition. (**D**) RAW 264.7 cells were infected with WT and ΔOmpA, at an MOI of 100, for 24 hours. The cells were then treated with chloroquine (20 µM) for 2 hours, following which they were harvested for Western blot analysis. The LC3-II and p62 levels were normalized to those of *β*-actin and are presented as mean ± SEM of three independent experiments. *P < 0.05 *versus* WT. WT, *Acinetobacter baumannii* ATCC 17978 wild-type; ΔOmpA, *A. baumannii* ATCC 17978 *ompA*-deletion mutant strain; ΔC, *A. baumannii* ATCC 17978 *ompA*-complemented strain, Ctrl, negative control.

In the next part of the study, we investigated whether exogenous OmpA from *A. baumannii* suppressed the AMPK pathway. To achieve this, RAW 264.7 cells were exposed to *A. baumannii*, recombinant AbOmpA (exogenous AbOmpA) protein, BML-275 (an AMPK inhibitor), SB-0206965 (an ULK inhibitor), or spautin-1 (which degrades the VPS34 PI 3-kinase complex). Exogenous AbOmpA effectively inhibited the phosphorylation of AMPK induced by each *Acinetobacter* strain (Fig. 3A through C; Fig. S1). Furthermore, BML-275 reduced the phosphorylation of downstream ULK Ser317, but not that of mTOR. These findings were similar to those obtained in the case of exogenous AbOmpA treatment, which notably inhibited the AMPK-ULK pathway (Fig. 3A through C; Fig. S1). This result suggests that AbOmpA inhibited the AMPK-ULK pathway during *Acinetobacter* infection. To assess the blockade of autophagic flux by exogenous AbOmpA, we evaluated the accumulation of LC3-II and p62 after treatment with chloroquine and exogenous AbOmpA. In the AbOmpA-mutant strain, the levels of LC3 and p62 decreased compared to that in the WT strain, due to autophagic degradation over a 24 hour infection period. However, exogenous AbOmpA increased the accumulation of LC3-II and p62 by inhibiting the autophagy-lysosomal fusion (Fig. 3D). In time-dependent infections, the AbOmpA-mutant strain exhibited a greater increase in LC3-II expression than the WT strain during the early stages of infection. However, after 12 hours of infection, the AbOmpA-mutant strain displayed reduced levels of LC3-II. The WT strain induced p62 and LC3 accumulation. Additionally, it was confirmed that changes in the levels of AMPK and Beclin-1 were evident from the early stage of infection. (Fig. S2). Although previous studies have reported that AbOmpA induces autophagy (17), we confirmed that both autophagy and apoptosis occur in a dose- and time-dependent manner. Administration of 1 µg of recombinant AbOmpA increased LC3 levels, whereas administration of 3 and 5 µg of recombinant AbOmpA triggered the production of LC3 and cleaved caspase 3 (Fig. S3). Notably, the WT strain induced apoptosis more significantly than the AbOmpA-mutant strain, through the activation of caspases-8,–9, and −3 (Fig. S4). These results suggest that exogenous AbOmpA protein plays a critical role in *A. baumannii*-induced apoptosis by inhibiting host defense mechanisms, especially autophagy.

**Fig 4:**
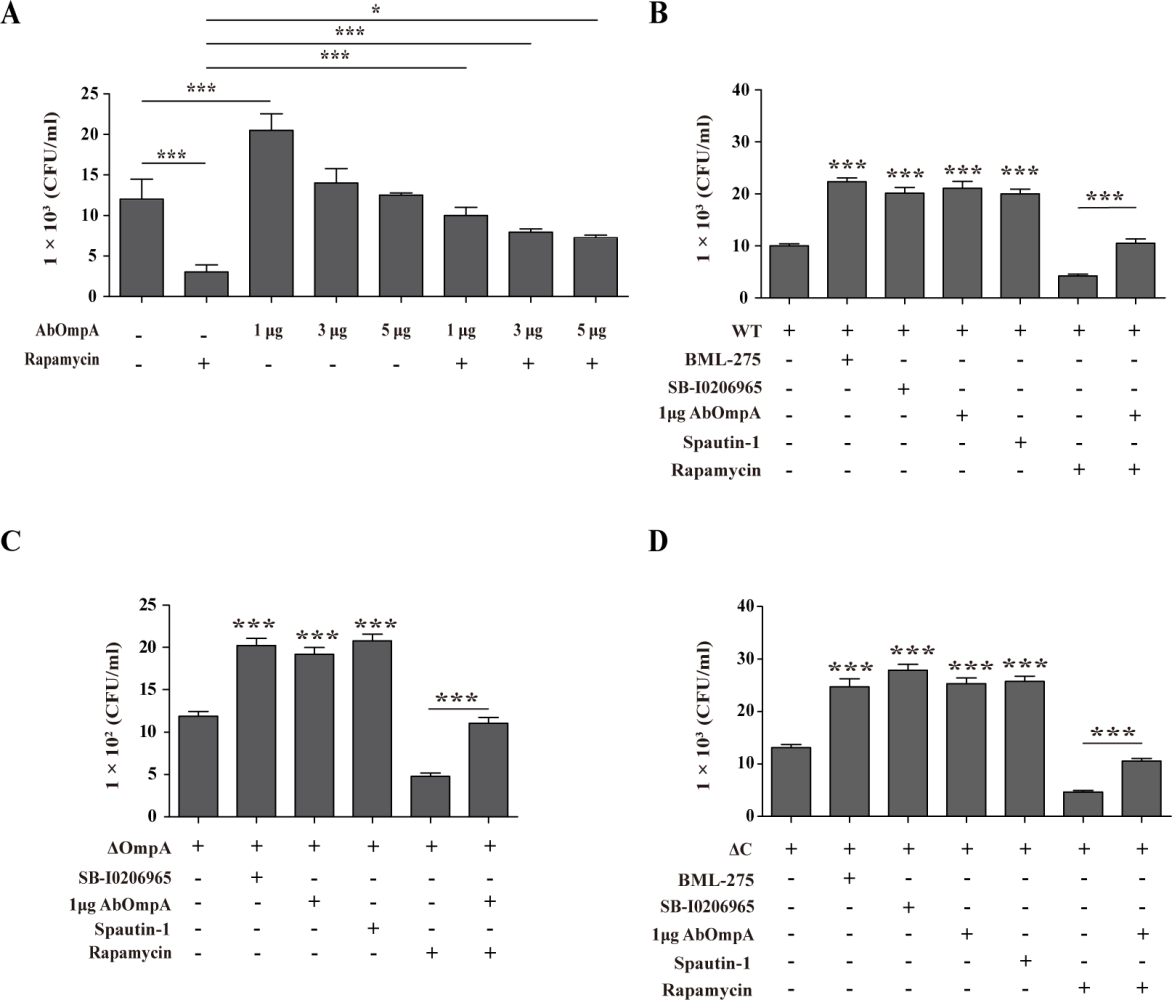
Exogenous AbOmpA increases intracellular *Acinetobacter baumannii*. (**A**) RAW 264.7 cells were infected with the wild-type strain at an MOI of 100, for 6 hours, and simultaneously treated with rapamycin and exogenous AbOmpA. (B–D) RAW 264.7 cells were infected with each of the *A. baumannii* strains at an MOI of 100, for 6 hours, and then simultaneously treated with BML-275 (AMPK inhibitor), SB-I0206965 (ULK inhibitor), spautin-1 (PI3 kinase class III complex inhibitor), exogenous AbOmpA, and/or rapamycin. Cells were treated for 1 hour in complete medium containing gentamicin. Data are presented as mean ± SEM of three independent experiments. ***P < 0.001 and *P < 0.05 *versus* infection control.

To elucidate bacterial invasion and clearance through autophagy, we conducted experiments using exogenous AbOmpA and various inhibitors to analyze the number of bacteria in cells. There was an increase in intracellular *A. baumannii* levels in cells treated with AbOmpA. However, as the concentration of exogenous AbOmpA increased, the number of bacteria gradually decreased within the cells (Fig. 4A). This led us to speculate that excessive induction of autophagy and apoptosis may account for this result. Interestingly, while rapamycin-induced autophagy decreased the number of intracellular bacteria, exogenous AbOmpA inhibited autophagy by rapamycin and increased the intracellular bacterial count (Fig. 4A). Furthermore, when we infected cells with each *Acinetobacter* strain and treated them with exogenous AbOmpA, we observed a similar increase in intracellular *Acinetobacter* numbers, as compared to the changes observed when autophagy was inhibited by BML-275, SB-0206965, and spautin-1 (Fig. 4B through D). These findings confirmed that AbOmpA hampers the initial autophagy mechanism and leads to an elevation in intracellular *A. baumannii* concentration.

**Fig 5:**
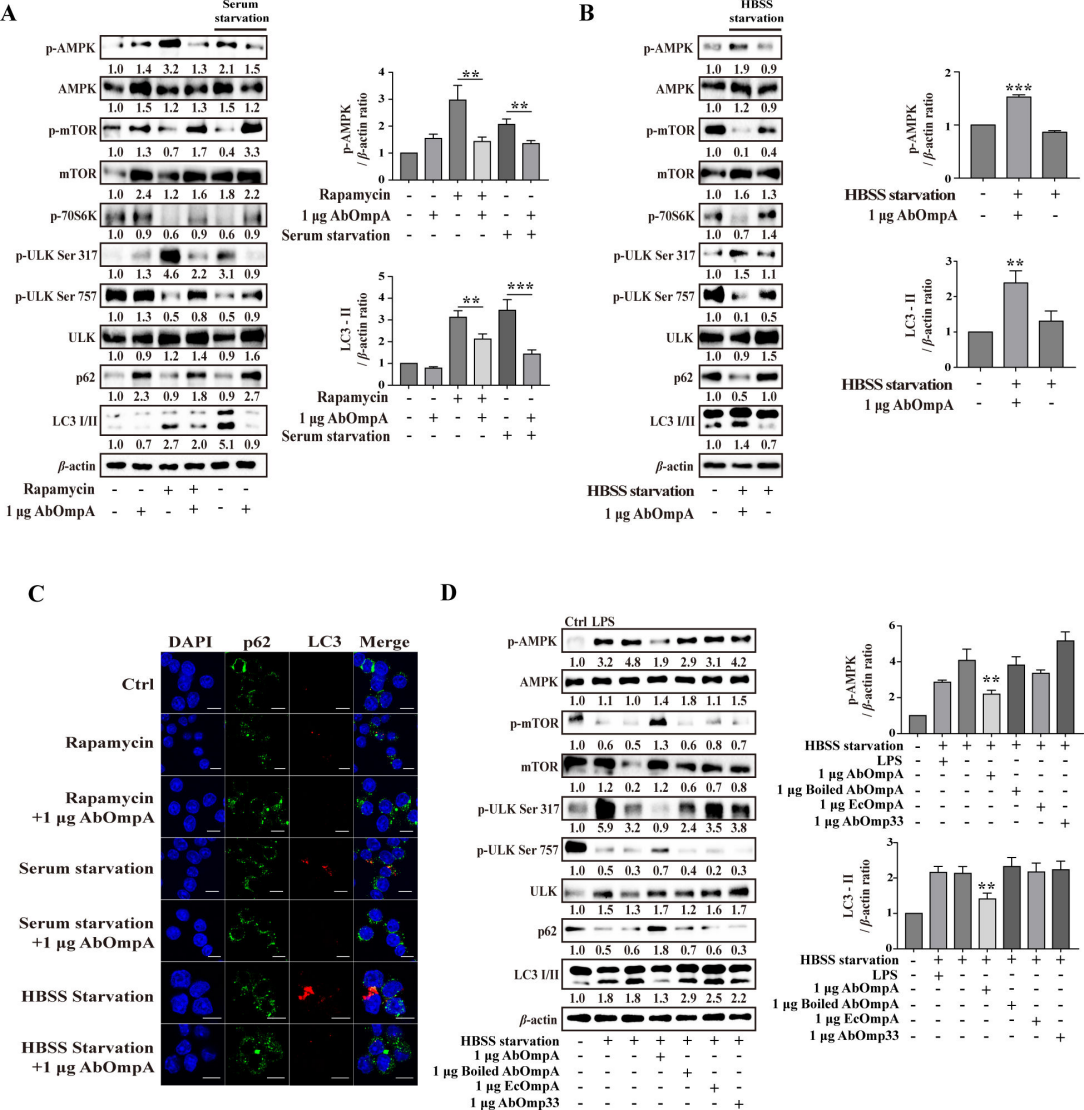
Exogenous AbOmpA suppresses autophagy under starvation conditions. (**A**) RAW 264.7 cells were subjected to serum-starvation (with Dulbecco’s modified Eagle medium without FBS) for 2 hours and rapamycin treatment for 2 hours. Target protein levels were normalized to those of *β*-actin and presented as mean ± SEM of three independent experiments. ***P < 0.001 and **P < 0.01. (**B**) Cells were subjected to HBSS-starvation, as described above, for 1 hour. All signal molecules were analyzed using Western blot. Target protein levels were normalized to those of *β*-actin and presented as mean ± SEM of three independent experiments. ***P < 0.001 and **P < 0.01 *versus* HBSS-starvation (**C**) LC3B (red) and p62 immunofluorescence (green) were monitored using confocal microscopy. Scale bar: 5 µm. The cells were treated with exogenous AbOmpA. (**D**) Cells were subjected to HBSS-starvation for 1 hour, as described above, and treated with LPS (100 ng/mL), exogenous AbOmpA (1 µg), exogenous AbOmp33 (1 µg), exogenous EcOmpA (1 µg)*,* or boiled exogenous AbOmpA (1 µg). The samples obtained after treatment were subjected to Western blot analysis. Target protein levels were normalized to those of *β*-actin and presented as mean ± SEM of three independent experiments. **P < 0.01 *versus* HBSS-starvation. Ctrl, negative control.

The autophagic response (specifically macroautophagy) is rapidly and strongly induced during Hank’s Balanced Salt Solution (HBSS)-starvation, owing to the lack of amino acids. Macroautophagy is regulated by AMPK and its interaction with the complex of ULK1 and mammalian TORC1 (mTORC1), an upstream regulator of autophagy, and other signaling pathways (33, 34). As illustrated in Fig. 5A, exogenous AbOmpA significantly suppressed the phosphorylation of AMPK during both serum starvation and rapamycin treatment, and decreasing LC3 and increasing p62 were observed. Exogenous AbOmpA substantially increased rapamycin-induced mTOR phosphorylation. Similar patterns were observed during HBSS-starvation (nutrient starvation), where the initial autophagy signaling involving AMPK, ULK, mTOR, and LC3-II was deactivated by exogenous AbOmpA and increased the p62 level (Fig. 5A and B; Fig. S5). The effect of exogenous AbOmpA on LC3-II downregulation was further confirmed using confocal imaging (Fig. 5C). Previous studies have reported the association of bacterial OmpA with mTORC2 and autophagic responses (35). Therefore, we conducted affinity purification of AbOmp33 and EcOmpA (Fig. S6) and treated cells with these proteins under HBSS starvation. Interestingly, EcOmpA, AbOmp33, and lipopolysaccharide (LPS) did not inhibit autophagy during HBSS-starvation. Moreover, the inhibition of autophagy was weakened when AbOmpA was denatured by means of boiling (Fig. 5D; Fig. S5). These data indicated that during HBSS-starvation, the autophagy suppression can solely be attributed to AbOmpA.

### Autophagy is suppressed by the interaction of Ca^2+^/calmodulin-dependent protein kinase 2 (CaMKK2) with AbOmpA, during *A. baumannii* infection

**Fig 6:**
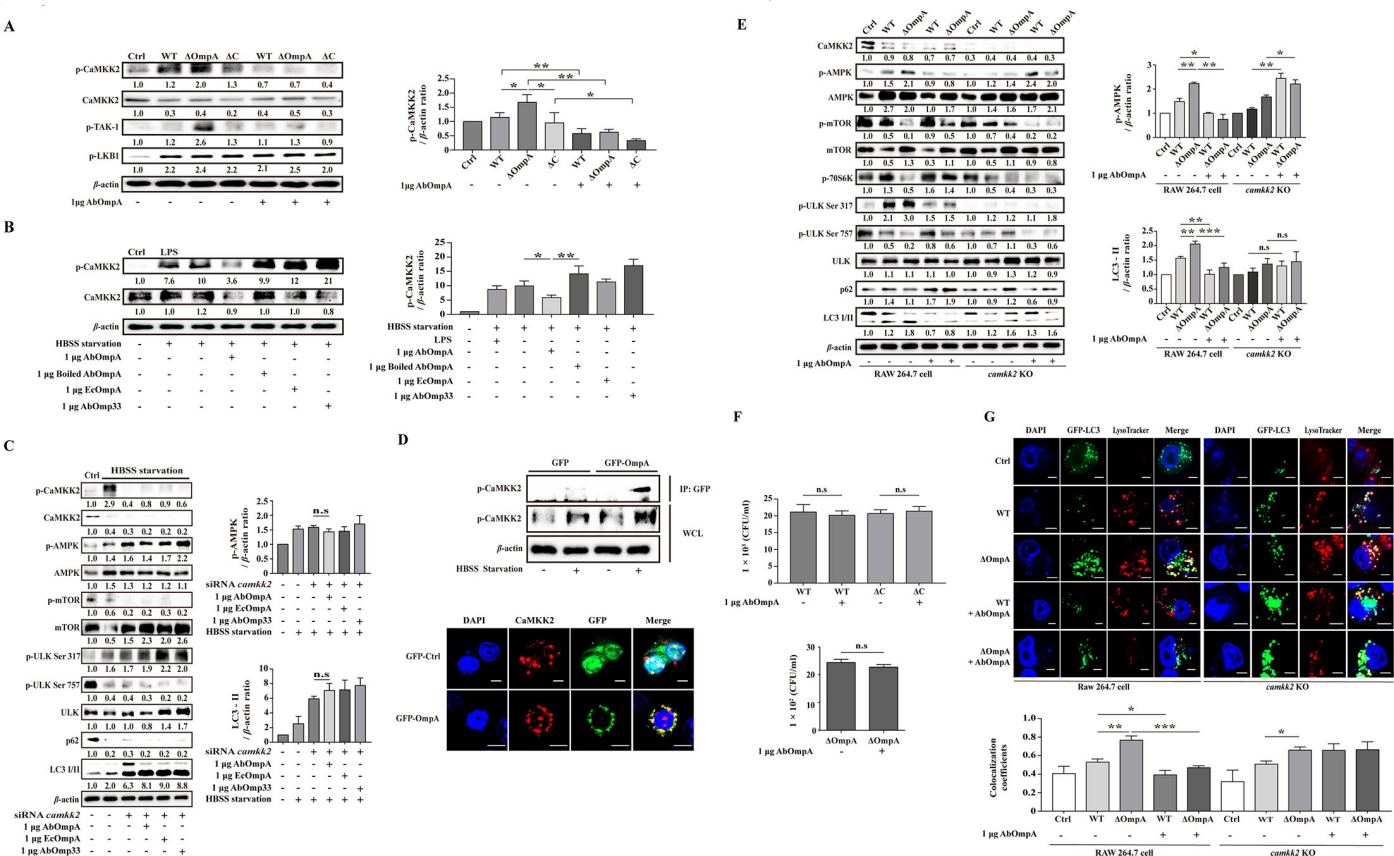
Exogenous AbOmpA interacts with CaMKK2. (**A**) RAW 264.7 cells were infected with WT, ΔOmpA, and ΔC at an MOI of 100, for 6 hours, and then treated with exogenous AbOmpA (1 µg). Target protein levels were normalized to those of *β*-actin and presented as mean ± SEM of three independent experiments. **P < 0.01 and *P < 0.05 (**B**) Cells were nutrient-starved for 1 hour and treated with LPS (100 ng/mL), exogenous AbOmpA (1 µg), exogenous AbOmp33 (1 µg), exogenous EcOmpA (1 µg), or boiled exogenous AbOmpA (1 µg). The samples obtained after treatment were subjected to Western blot analysis. Target protein levels were normalized to those of *β*-actin and presented as mean ± SEM of three independent experiments. **P < 0.01 and *P < 0.05 (**C**) RAW 264.7 cells were transfected with a non-silencing siRNA (NT) and *camkk2*-targeting siRNA, subjected to HBSS-starvation for 1 hour, as described above, and treated with exogenous AbOmpA (1 µg), exogenous AbOmp33 (1 µg), or exogenous EcOmpA (1 µg). The samples obtained after treatment were subjected to Western blot analysis. Target protein levels were normalized to those of *β*-actin and presented as mean ± SEM of three independent experiments. (**D**) RAW 264.7 cells were transfected with GFP-AbOmpA and then subjected to HBSS-starvation for 1 hour, as described above. The cell lysates obtained were immunoprecipitated using anti-GFP and analyzed by means of Western blot using anti-p-CaMKK2. Representative confocal images of CaMKK2 and GFP. Scale bar: 5 µm. (**E**) RAW 264.7 and *camkk2*-knockout cells were infected with WT, ΔOmpA, and ΔC, at an MOI of 100, for 6 hours, and treated with exogenous AbOmpA (1 µg). The samples obtained after treatment were subjected to western blot analysis. Target proteins levels were normalized to those of *β*-actin and presented as mean ± SEM of three independent experiments. ***P < 0.001, **P < 0.01, and *P < 0.05 (**F**) RAW 264.7 and *camkk2*-knockout cells were infected with WT, ΔOmpA, and ΔC, at an MOI of 100, for 6 hours, and treated with exogenous AbOmpA (1 µg). Cells were treated for 1 hour in complete medium containing gentamicin. Data are presented as mean ± SEM of three independent experiments. (**G**) Representative confocal images of LC3 (GFP-LC3) and LysoTracker, after infection for 6 hours. Scale bar: 5 µm. Pearson’s correlation coefficient was calculated using JACoP (*n* = 5). Data are presented as mean ± SEM. ***P < 0.001, **P < 0.01, and *P < 0.05. WT, *Acinetobacter baumannii* ATCC 17978 wild-type; ΔOmpA, *A. baumannii* ATCC 17978 *ompA*-deletion mutant strain; ΔC, *A. baumannii* ATCC 17978 *ompA*-complemented strain; Ctrl, negative control.

AMPK plays a crucial role in the initial regulation of autophagy and is activated by phosphorylation. There are three main mechanisms for inducing AMPK phosphorylation: *via* LKB1, CaMKK2, and transforming growth factor-β-activated kinase 1 (TAK-1) (19, 36, 37). Given the effects of AbOmpA treatment on the AMPK pathway and downstream signaling, we investigated the effect of AbOmpA on the activation of LKB1, CaMKK2, and TAK-1, which are upstream kinases that activate AMPK Thr172. As depicted in Fig. 6A, LKB1 phosphorylation was induced by *A. baumannii* infection, but not affected by exogenous AbOmpA. However, the AbOmpA-mutant strain exhibited higher activation of TAK-1 and CaMKK2 phosphorylation than the WT and complemented strains. Additionally, exogenous AbOmpA suppressed the phosphorylation of TAK-1 and CaMKK2 during *A. baumannii* infection. To determine the specific effect of AbOmpA on CaMKK2, as compared to those of the other OMPs, we treated cells with each OMP under the HBSS-starvation condition. Phosphorylation of CaMKK2 was decreased by AbOmpA, but not LPS, EcOmpA, or AbOmp33 (Fig. 6B).

To analyze the interaction between CaMKK2 and AbOmpA, we used small interfering RNA (siRNA) to silence *camkk2*, with cells expressing nontarget siRNA used as a control. Under HBSS-starvation conditions, we examined the effects of AbOmpA, AbOmp33, and EcOmpA on autophagy signaling. In CaMKK2-silenced cells, treatment with AbOmpA, AbOmp33, or EcOmpA did not influence LC3-II levels or the phosphorylation of AMPK and ULK Ser317. Furthermore, there was no increase in the phosphorylation of mTOR and ULK Ser757 (Fig. 6C; Fig. S7). We then performed co-immunoprecipitation (Co-IP) experiments with CaMKK2 and AbOmpA under HBSS-starvation conditions, which revealed an interaction between AbOmpA and CaMKK2 in the cytosol (Fig 6D; Fig. S8). Consistently, the imaging data also showed an interaction between AbOmpA and CaMKK2 (Fig. 6D). The exogenous protein for AbOmpA and AbOmp33 was stained with His-tag and imaged together with CaMKK2. Only AbOmpA showed colocalization with intracellular CaMKK2 (Fig. S9). Moreover, we employed the CRISPR–Cas9 system to investigate the CaMKK2–AbOmpA interaction. For this, we first knocked out CaMKK2 in RAW 264.7 cells (Fig. S10). Under HBSS-starvation conditions, we confirmed that AbOmpA had no influence on *camkk2*-knockout cells, as compared to that on WT-RAW 264.7 cells (Fig. S10). In cells infected with the WT and AbOmpA-mutant strains, the inhibitory effect of exogenous AbOmpA on the autophagy pathway was not observed in *camkk2*-knockout cells, as compared to that in WT RAW 264.7 cells (Fig. 6E; Fig. S7). Infection with each *A. baumannii* strain resulted in increased intracellular counts in the *camkk2*-knockout cells, with no significant variation in intracellular bacterial count by exogenous AbOmpA (Fig. 6F). To further clarify the LC3 levels and autophagosome generation, we pretreated cells with GFP-LC3 and labeled lysosomes with LysoTracker. As shown in Fig. 6G, exogenous AbOmpA inhibited LC3 levels and lysosomal colocalization in infected cells. However, the inhibitory effect of exogenous AbOmpA was not observed in *camkk2*-knockout cells. These data indicated that AbOmpA interacts with CaMKK2 to inhibit autophagy.

**Fig 7:**
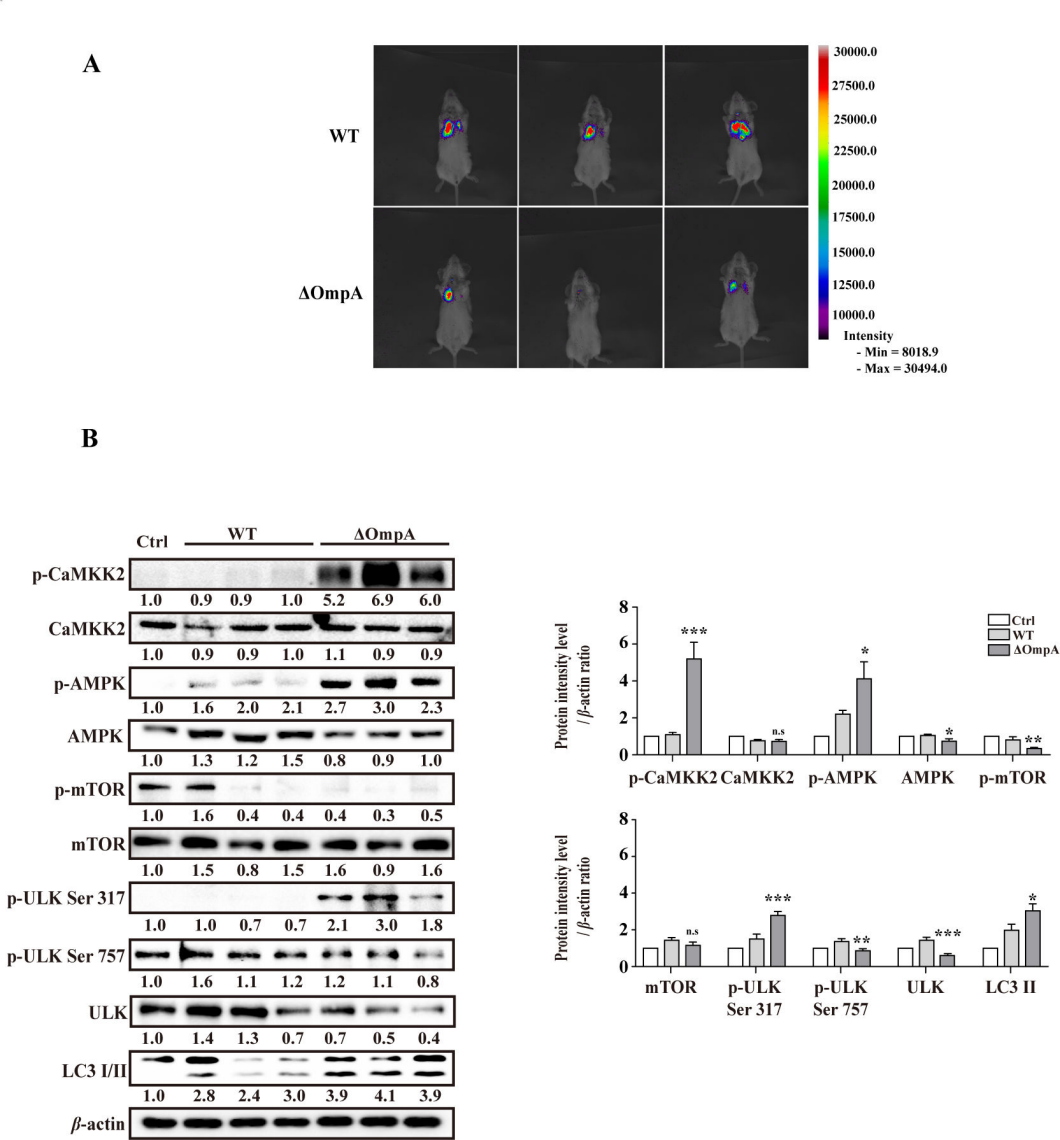
AbOmpA regulates autophagy *in vivo*. (**A**) BALB/c mice were intratracheally infected with each of the bioluminescent *Acinetobacter baumannii* strains. The IVIS images indicate the intensity of luminescence. Representative images indicate bacterial challenge for 1 day. (**B**) Lung tissues from the infected mice were lysed and analyzed using Western blot. Target protein levels were normalized to those of *β*-actin ratios (*n* = 3). Data are presented as mean ± SEM. ***P < 0.001, **P < 0.01, and *P < 0.05 *versus* WT. Ctrl, negative control.

### AbOmpA-mediated autophagy is critical for *A. baumannii* infection in the lung tissues

Previous studies have established a correlation between OmpA of *A. baumannii* and mTOR signaling pathways in the lungs of SD rats, thereby highlighting its significance in bacterial colonization and dissemination *in vivo* (14). To gain further insights, we investigated the involvement of the CaMKK2 and AMPK pathways in a mouse model. Intratracheal infection was induced in BALB/c mice using bioluminescent *A. baumannii* strains. One day after infection, we measured the bioluminescence emitted by the WT and AbOmpA-mutant strains within the lungs. Remarkably, the AbOmpA-mutant strain exhibited reduced bioluminescence in the lung spaces, as compared to the WT strain (Fig. 7A). Subsequently, the mice that underwent bioluminescence measurements were sacrificed to examine the autophagy signals. In the lung tissue, the AbOmpA-mutant strain displayed significantly higher phosphorylation levels of CaMKK2, AMPK, and ULK Ser317 than the WT strain (Fig. 7B). Moreover, the AbOmpA-mutant strain-infected mice displayed more pronounced expression of LC3-II (an indicator of autophagy activation) in the lungs than the WT strain-infected mice (Fig. 7B). Collectively, these findings suggested that OmpA of *A. baumannii* evades the host immune defense system by inhibiting autophagy in the lung tissue.

## DISCUSSION

In the current study, we found that OmpA in *A. baumannii* inhibits the phosphorylation of CaMKK2, which regulates the downstream activation of AMPK, mTOR, and ULK1. This causes a decrease in autophagy signaling due to declining phosphorylation of AMPK and ULK1. Additionally, under starvation conditions, AbOmpA suppressed the phosphorylation of AMPK and CaMKK2.

Bacteria (intra- and extracellular) can survive by impeding autophagy mechanisms or circumventing the immune system (24). Specifically, during the autophagy initiation phase, *Salmonella* Typhimurium inhibits AMPK activation while activating mTOR, thereby allowing the bacteria to rapidly overcome autophagy in macrophages (38). The extracellular bacterium enteropathogenic *E. coli* utilizes the effector EspG, which contains a GTPase-activating protein domain, to hinder autophagy induction by rendering Rab1 inactive (24). In *S. flexneri* infections, OspB activates mTORC1, resulting in the suppression of autophagy (39). According to a recent study, the OmpA of *A. baumannii* triggers autophagy through the MAPK/JNK signaling pathway in HeLa cells and mTOR signaling pathway in the lungs of SD rats. In addition, An *et al*. found that AbOmpA induces incomplete autophagy in HeLa cells (14, 17). We found that the AbOmpA-mutant strain was more potent in inducing autophagy than the WT strain during early infection. It also included a comparative analysis between the WT and AbOmpA-mutant strains at both the early and late stages of infection. AbOmpA inhibits autophagosome–lysosome fusion to a degree similar to that observed with chloroquine treatment. Based on these results, we suggest that AbOmpA inhibits the autophagy process during *A. baumannii* infection.

Several studies have demonstrated a relationship between OMPs, autophagy, and apoptosis. For instance, *A. baumannii* Omp33-36 protein has been shown to induce apoptosis and block autophagy, leading to accumulation of autophagosomes. Omp33-36-containing OMVs from *A. baumannii* have been shown to cause the accumulation of LC3B-II and p62 (16). In contrast, EcOmpA and mitochondrial OMPs stimulate degradation of LC3B and p62 through the mTORC2-dependent autophagy pathway (35). OMVs of *P. aeruginosa* and *H. pylori* induce autophagy in epithelial cells (40). *Listeria monocytogenes* OMVs inhibit the purified LLO-mediated autophagy response, by blocking AMPK activation and LC3 lipidation, while increasing mTOR phosphorylation; moreover, the addition of OMVs to mouse embryonic fibroblasts increases intracellular *L. monocytogenes* (41). ExoS from *P. aeruginosa* and CagA from *H. pylori* inhibit autophagy induction by regulating the mTOR signaling pathway (26, 42). The OMVs of *A. baumannii* contain OmpA, proteases, phospholipases, and catalases (43). We demonstrated that exogenous AbOmpA inhibited autophagy by suppressing AMPK phosphorylation at Thr172 and ULK1 phosphorylation at Ser317, while inducing mTOR under both infection and starvation stress conditions. Previous studies have demonstrated that host cell death is induced when AbOmpA is treated at concentration of 5 µg/mL or higher (44). Furthermore, treatment with 10 µg/mL induces both autophagy and apoptosis (17). As shown in Fig. 4A and 5A, low concentrations of AbOmpA effectively inhibit rapamycin-induced autophagy and increase intracellular bacteria counts. However, as the treatment concentration increases, a slight reduction in this increase is observed, with the intracellular bacterial count at 5 µg/mL resembling that observed under normal infection conditions. This suggests an interaction between apoptosis and autophagy. It can be speculated that excessive autophagy activation, along with AbOmpA-induced cytochrome c release and caspase-9 activation, contributes to autophagy inhibition. Caspases regulate both apoptosis and autophagy, with caspase-9 interacting with ATG7 and caspase 8 with ATG3 (45). Based on these observations, we propose that low concentrations of AbOmpA effectively inhibit the initiation phase of autophagy by interfering with the phosphorylation process. Importantly, this study primarily focused on the effects of AbOmpA at low concentrations to exclude outcomes influenced by apoptosis. In the present study, AbOmpA suppresses autophagy induction, whereas AbOmp33 and EcOmpA do not. Based on our findings, we suggest that the presence of AbOmpA within *A. baumannii* OMVs may contribute to the accumulation of LC3B-II and p62. Our results are consistent with those of other studies showing inhibition of early autophagy mechanisms by bacterial proteins or toxins. Notably, inhibition of autophagy by AbOmpA increased the intracellular survival of bacteria, a finding consistent with those of previous bacterial studies. These results suggest that the inhibition of autophagy by AbOmpA contributes to bacteria survival, leading to apoptosis after invasion.

CaMKK2 has a significant impact on various physiological processes related to energy and glucose metabolism, inflammation, and cancer, by influencing signaling cascades and the pathogenesis of metabolic diseases (46). LKB1 acts as a master upstream kinase and metabolic sensor that affects the immune response and directly activates AMPK, an energy sensor (47). Phosphorylation of the AMPK catalytic subunit (α) at Thr172 is induced by the phosphorylation of CaMKK2 and LKB1 (13). LKB1-deficient mice show impaired host immune responses during pneumonia (48). *Salmonella* Pathogenicity Island 2-encoded virulence factors impair the Sirt1/LKB1/AMPK complex (19). Although our study found that *A. baumannii* infection induced LKB1 phosphorylation, AbOmpA had no effect on LKB1. Previous research showed that CaMKK2 is activated by *E. coli*-induced cytosolic calcium efflux, leading to the phosphorylation of AMPK, and that *camkk2* deficiency increases the intracellular *E. coli* load by autophagy inhibition (49). In this study, we observed that the AbOmpA-mutant strain induced higher CaMKK2 phosphorylation than that observed in the WT and complemented strains *in vitro* and *in vivo*. Exogenous AbOmpA treatment inhibited the increased phosphorylation of CaMKK2 by each *A. baumannii* strain *in vitro* and also inhibited CaMKK2 phosphorylation under starvation conditions. Inhibition of autophagy induction by AbOmpA was diminished by silencing-mediated *camkk2* knockdown. Our data indicated that AbOmpA interacted with CaMKK2 and inhibited the autophagy mechanism, but there were no significant changes in autophagy protein levels upon AbOmpA treatment in the *camkk*2-knockout RAW 264.7 cell line infected with *A. baumannii*. Therefore, we concluded that AbOmpA inhibits autophagy via interaction with CaMKK2.

Our study provides a different perspective on AbOmpA, which has previously been reported to induce autophagy and apoptosis in HeLa cells (17). We demonstrated that low levels of AbOmpA inhibit autophagy by suppressing CaMKK2 phosphorylation. However, there were limitations as we did not delve into the connection between apoptosis, autophagy, or other regulated cell death mechanisms. Future research should concentrate on AbOmpA’s structure, especially the specific region responsible for this interaction. In conclusion, our findings reveal a novel function of AbOmpA in suppressing CaMKK2. This particular inhibitory effect sheds light on the modulation of autophagy and the enhanced survival mechanisms post an *A. baumannii* infection. This knowledge holds potential relevance for other extracellular bacterial infections, paving the path for therapeutic strategies targeting both AbOmpA and *A. baumannii*.

## MATERIALS AND METHODS

### Bacterial strains

The *A. baumannii* ATCC 17978 WT, *A. baumannii* ATCC *ompA*-deletion mutant (ΔOmpA), *ompA*-complemented strains of the Δ*ompA* mutant of ATCC 17978 (ΔC), and bioluminescent strains were grown separately in Luria–Bertani broth, containing 1.5% (wt/vol) agar, at 37°C. The ΔOmpA, ΔC, and bioluminescent strains were constructed using a conjugation method (50, 51).

### Cell line and reagents

RAW 264.7 cells (*Mus musculus* macrophage TIB-71; ATCC) were cultured in Dulbecco’s modified Eagle medium (DMEM; WELGENE, LM001-05) supplemented with 10% fetal bovine serum (FBS; WELGENE, S001-01) and 1% antibiotic–antimycotic solution (Welgene; LS203-01), at 37°C, in an atmosphere containing 5% CO_2_. The cells were treated with the inhibitors 1 µM BML-275 (Santa Cruz Biotechnology, sc-200689), 1 µM SB-I0206965 (Cayman Chemicals, 18477), 40 µM spautin-1 (Sigma, SML0440), or 6 µM rapamycin (Santa Cruz Biotechnology, sc-3504); then infected with *A. baumannii*; and maintained throughout the course of the experiment. For starvation, the cells were cultured in Hank’s balanced salt solution (HBSS; Sigma, H6648) or Dulbecco’s modified Eagle medium and treated with 100 ng/mL LPS-EB (from *E. coli* O111:B4 [Invivogen, TLRL-EBLPS]) and 1 µg of each AbOmpA during the course of the experiment. To inhibit the autophagosome fusion of the infected RAW 264.7 cells, 20 µM chloroquine diphosphate salt (Sigma, C6628) was added 2 hours before the end of the experiment, and 200 nM bafilomycin A1 (Invivogen, tlrl-baf1) treatment was added 4 hours before the end of the experiment, and then the cells were harvested.

### Immunoblotting analysis

RAW 264.7 cells were washed with cold Dulbecco’s phosphate-buffered saline (DPBS; WELGENE, LB001-02), and the entire population was extracted and lysed using radioimmunoprecipitation assay lysis buffer and protease inhibitor cocktail (Thermo Scientific, 78429) for 15 minutes, at 4°C. The cell lysates were centrifuged at 14,000 × *g* for 15 minutes, at 4°C, and quantified using Bradford assay (Bio-Rad, 5000006). Quantified proteins (20 or 50 µg) were separated using 8% or 15% sodium dodecyl sulfate-polyacrylamide gel electrophoresis. The separated proteins were electrotransferred onto a polyvinylidene fluoride membrane (EMD Millipore, IPVH00010). The membranes were then blocked with EZBlock Chemi (ATTO, AE-1475) and incubated with diluted 1 :1,000 primary antibodies. The following antibodies were used: rabbit anti-p-AMPK (Cell Signaling Technology, 2535), rabbit anti-p-mTOR (Cell Signaling Technology, 2971), rabbit anti-p-70 S6K (Cell Signaling Technology, 9205), rabbit anti-p-ULK Ser757 (Cell Signaling Technology, 6888), rabbit anti-SQSTM1/p62 (Sigma, P0067), mouse anti-LC3-I/II (Cell Signaling Technology, 83506), rabbit anti-AMPK (Cell Signaling Technology, 2532), rabbit anti-mTOR (Cell Signaling Technology, 2972), rabbit anti-ULK (Cell Signaling Technology, 8054), rabbit anti-ATG16L1 (Cell Signaling Technology, 8089), rabbit anti-p-ATG16L1 (Abcam, ab195242), rabbit anti-ATG12 (Cell Signaling Technology, 4180), rabbit anti-p-Beclin-1 (Cell Signaling Technology, 84966), rabbit anti-caspase 3 (Cell Signaling Technology, 9662), rabbit anti-caspase 8 (Santa Cruz Biotechnology, sc-81656), mouse anti-caspase 9 (Cell Signaling Technology, 9508), rabbit anti-p-CaMKK2 (Cell Signaling Technology, 12818), rabbit anti-p-LKB1 (Cell Signaling Technology, 3482), rabbit anti-β-actin (Cell Signaling Technology, 4970), rabbit anti-p-ULK Ser317 (Invitrogen, PA5-104556), rabbit anti-CaMKK2 (Invitrogen, PA5-102029), rabbit anti-ATG5 (Cell Signaling Technology, 12994), rabbit anti-p-TAK1 (Invitrogen, MA5-15073), mouse anti-Beclin-1 (Santa Cruz Biotechnology, sc-48341), and mouse PI 3-kinase p100 (Santa Cruz Biotechnology, sc-365404) at 4°C overnight. The blots were further incubated with the following appropriate horseradish peroxidase-conjugated secondary antibodies: Goat Anti-Rabbit IgG Antibody, Peroxidase-Conjugated (Merck Millipore 401353) and Goat Anti-Mouse IgG Antibody, and (H + L) HRP conjugate (Merck Millipore, 401215). The membranes were subjected to enhanced chemiluminescence (WesternBright ECL kit; Advanstar, K-12045), according to the manufacturer’s instructions, and visualized using a ChemiDoc XRS imaging system (Bio-Rad, Richmond, CA, USA).

### Purification of *A. baumannii* AbOmpA, AbOmp33, and *E. coli* OmpA

The *A. baumannii* ATCC 17978 *ompA* gene with *BamH*I (New England Biolabs, R0136) and *Hind*III (New England Biolabs, R3104) sites and *omp33* gene with *BamH*I and *Sac*I (New England Biolabs, R3156) sites were amplified using PCR (Table S1). The PCR products obtained were digested and ligated into pET22b (+). The expression plasmid obtained was then transformed into *E. coli* DH5α (Real Biotech Corporation, RH617) and BL21 cells (Real Biotech Corporation, RH217). Protein overexpression in the cultured *E. coli* BL21 cells was induced using IPTG (LPS Solution, IPTG005). The collected cells were sonicated and then centrifuged at 12,000 rpm, 4°C, for 20 minutes. The supernatant obtained was collected and subjected to soluble protein purification using Ni-NTA affinity chromatography (Qiagen, 30210), as previously described (52). The *E. coli* strain CFT073 *ompA* gene with *EcoR*I (New England Biolabs, R3101) and *Hind*III sites was amplified using PCR. The PCR products were digested and ligated into pET22b (+). Protein overexpression in the cultured *E. coli* BL21 cells was induced using IPTG. The cultured cells were sonicated and then subjected to centrifugation at 8,000 rpm, 4°C, for 20 minutes. To collect the clear supernatant from the insoluble protein, the pellet was sonicated using binding buffer (20 mM Tris [pH 8.0], 500 mM NaCl, and 5 mM imidazole), centrifuged, sonicated using a binding buffer containing 6 M urea, and centrifuged again (14,000 × *g*, 4°C, for 20 minutes). The obtained supernatant was applied to an Ni-NTA affinity column. The column was then washed with a binding buffer containing 6 M urea and washing buffer (20 mM Tris [pH 8.0], 500 mM NaCl, 60 mM imidazole, and 6 M urea). The protein was eluted in elution buffer (20 mM Tris pH 8.0, 500 mM NaCl, 100 mM imidazole, and 6 M urea). All purified proteins were dialyzed against 50 mmol/L Tris-HCl, 100 mM NaCl [pH 7.6], at 4°C, for 1 day; concentrated through a Amicon Ultra centrifugal filter (30,000 Da molecular weight cut-off, Merck Millipore, UFC903024); and sterilized using a 0.2 µm filter. The protein concentration was determined using the Pierce BCA Protein Assay Kit (Thermo Scientific, 23227). The purified proteins were stored at −80°C until use.

### Bacterial invasion assay

RAW 264.7 cells were plated into 12-well plates, at a density of 2 × 10^5^ cells/well, and treated with the inhibitors, recombinant AbOmpA, or/and *A. baumannii*. The cells were washed three times with PBS and incubated with Dulbecco’s modified Eagle medium containing 10% fetal bovine serum and 300 µg/mL of gentamicin, for 1 hours, to kill all external bacteria. The cells were then washed three times and lysed with 0.1% Triton X-100 (Sigma, T8787). Dilutions from each well were plated on LB agar plates, and the colonies were counted to quantify the bacteria that survived intracellularly.

### Visualization of acridine orange and monodansylcadaverine staining

RAW 264.7 cells were seeded into 12-well plates with sterile circular 18 mm cover slips and treated with rapamycin and *A. baumannii* strains. Following that, the cells were incubated with 1 µg/mL acridine orange (Invitrogen, A1301), at 37°C, for 30 minutes. The stained cells were visualized under a fluorescence microscope (BX53 with an attached DP70 camera, Olympus, Tokyo, Japan). For flow cytometric analysis, the infected cells were harvested, incubated with 1 µg/mL acridine orange, at 37°C, for 30 minutes, or 50 µM monodansylcadaverine (Sigma, D4008), at 37°C, for 10 minutes, and then fixed with 4% paraformaldehyde. The stained cells were analyzed using a NovoCyte Flow Cytometer (ACEA Biosciences Inc., San Diego, CA, USA), and the percentage of cells was analyzed using the FlowJo software (Tree Star).

### Autophagosome detection using transmission electron microscopy

RAW 264.7 cells were seeded at a density of 3 × 10^5^ cells into 12-well culture plates and then stimulated with *A. baumannii* strains. After 6 hours of infection, the collected cells were incubated with 2.5% glutaraldehyde, at 4°C, for 2 hours, and washed three times with PBS. The cells were then subjected to electron microscopy processing, as described previously (53). Briefly, the cells were fixed with 1% osmium tetroxide at room temperature, for 2 hours, and washed three times with PBS. The cells were dehydrated and infiltrated with an ethanol and propylene oxide series. The cells embedded in Epon812 were cut into ultrathin sections (70 nm thickness) using an ultra-microtome (Ultracut UCT, Leica, Microsystems, Wetzlar, Germany, installed at the Korea Basic Science Institute), which were collected on 100-mesh copper grids. After staining with 2% uranyl acetate and lead citrate, the sections were analyzed using a transmission electron microscope (JEM-2010, JEOL Ltd, Tokyo, Japan) installed at the Center for University-Wide Research Facilities at Chonbuk National University (Chonbuk, South Korea).

### Co-IP analysis

RAW 264.7 cells were seeded into 12-well plates and upon reaching 70% confluence transiently transfected with plasmids expressing pEGFP-C1-*ompA* (Table S1) and pEGFP-C1 (Addgene, 6084-1) using Lipofectamine 3000 (Invitrogen, L3000001), according to the manufacturer’s instructions. The cells were then harvested and lysed in a lysis/equilibration buffer containing protease inhibitors. After incubation on ice for 15 minutes, the cell lysates were collected and centrifuged at 17,000 × *g*, 4°C for 10 minutes and then incubated with a monoclonal mouse anti-GFP antibody (1 mg/mL; Invitrogen, MA5-15256) for up to 60 minutes, at 4°C. Co-IP was performed using a Capturem IP & Co-IP kit (TaKaRa, 635721), according to the manufacturer’s instructions. The eluted samples were separated using sodium dodecyl sulfate-polyacrylamide gel electrophoresis and analyzed using Western blot.

### Immunofluorescence microscopy

RAW 264.7 cells were seeded into 12-well plates with sterile circular 18 mm cover slips. The treated and transfected cells were washed with PBS, fixed with 4% paraformaldehyde for 10 minutes, and permeabilized with 0.1% Triton X-100 for 5 minutes. The cells were incubated with 1% bovine serum albumin for 1 hour, followed by incubation with 1:100~200 diluted rabbit anti-SQSTM1/p62 (Sigma, P0067), rabbit anti-LC3-I/II (Cell Signaling Technology, 2775), mouse anti-LC3-I/II (Cell Signaling Technology, 83506), rabbit anti-CaMKK2 (Invitrogen, PA5-102029), mouse anti-His-Tag (Santa Cruz Biotechnology, sc-8036), or mouse anti-LAMP2 monoclonal antibody H4B4 (Invitrogen, MA1-205) in 1% bovine serum albumin, at 4°C overnight. The cells were washed with PBS and then incubated with Alexa Fluor-conjugated secondary antibodies for 1 hour, at room temperature. The following antibodies were used: Alexa Fluor 488-conjugated F(ab′)2 fragment of goat anti-rabbit or mouse IgG (Cell Signaling Technology, 4412 and 4408) and Alexa Fluor 594-conjugated F(ab′)2 fragment of goat anti-rabbit or mouse IgG (Cell Signaling Technology, 8889 and 8890). For autophagy flux monitoring, the cells were transfected with pMRX-IP-GFP-LC3-RFP-LC3ΔG (Addgene, #84572) using Lipofectamine 3000. For lysosome and LC3 fusion detection, the cells were transfected with pEGFP-C1-LC3 (Table S1) using Lipofectamine 3000, and the transfected cells were incubated with LysoTracker Deep Red (100 nM for 30 minutes; Invitrogen, L12492). The cells were mounted onto microscope slides using a mounting medium containing 4′,6-diamidino-2-phenylindole (VECTASHIELD, H-1200). Fluorescence images were captured using a confocal microscope (DMi8 confocal microscope, Leica Microsystems, Germany) and LSM 900 confocal microscope (Zeiss, Germany) with ZEN software (Zeiss), Adobe Illustrator, and Fiji software.

### Transfection

siRNAs and nonsilencing siRNAs (negative controls) were synthesized by Bioneer (South Korea). The sequence of the siRNA against mouse *camkk2* was 5′-CAGCUAAGCUAAUUCUCUU-3′. RAW 264.7 cells were cultured at 80% confluency in 12-well plates and transfected with plasmids using the Lipofectamine RNAiMAX mixture (Invitrogen, 13778075), as per the protocol provided by the manufacturer. After incubation for an additional 48 hours, the transfected cells were used for each experiment.

### CaMKK2 deletion using CRISPR/Cas9

Deletion of the *camkk2* gene in RAW 264.7 cells was carried out using the CRISPR/Cas9 system. Guide RNAs (gRNAs) consisting of sequences that both interface with the Cas protein and guide the complex to the *camkk2* target gene were selected using the web tool CHOPCHOP (https://chopchop.cbu.uib.no/). The gRNA sequence used for *camkk2* was (5′-CCACGTCTCCATTACCGGTTTGCA-3′). Selected gRNA oligos were inserted into the px330-puro vector, as described previously (54). RAW 264.7 cells were transfected with a vector with gRNA targeting using Lipofectamine 3000 as per the manufacturer’s protocol. After 24 hours, the cells were incubated with 5 µg/mL puromycin for 2 days. Single clones were selected, cultured, and confirmed by means of immunoblotting and DNA sequencing.

### Animal experiments

Seven-week-old female BALB/C mice were purchased from Nara Biotech and maintained under specific pathogen-free conditions, in accordance with the guidelines of the Korean Food and Drug Administration and the Institutional Animal Care and Use Committee of Chungnam National University (permission number: 202112A-CNU-206). Neutropenia was induced in the mice by means of intraperitoneal injections of cyclophosphamide (150 mg/kg [Sigma, C7397]), on day 2 prior to the injection of bacterial cells. The mice were anesthetized with tribromoethanol, and groups of five were injected intratracheally with 50 µL of 1.5 × 10^8^ colony-forming units/mL of bacteria. The control mice groups were injected with 50 µL of sterilized PBS (pH 7.4). The mice were euthanized 1 day after bacterial challenge. To investigate the luminescence intensity, the mice were imaged using an *in vivo* imaging system (VISQUE InVivo Smart-LF, Animalab, Vieworks, Anyang, South Korea). The lungs of the mice were washed with sterile PBS and homogenized using radioimmunoprecipitation assay lysis buffer (Cell Signaling Technology, 9806). Tissue samples were separated using sodium dodecyl sulfate-polyacrylamide gel electrophoresis and Western blot.

### Statistical analysis

The results have been expressed as mean and standard error of mean of at least three independent experiments. All data were analyzed using unpaired Student’s *t*-tests or a one-way analysis of variance, followed by Tukey’s multiple comparisons test, as appropriate, using Prism (GraphPad Software). Differences between experimental groups were considered significant at *P < 0.05, **P < 0.01, and ***P < 0.001.

## Data Availability

This study includes no data deposited in external repositories.

## AFTER EPUB

*[This article was published on 25 February 2025 with errors in the affiliations and Materials and Methods. The errors were corrected in the current version, posted on 3 March 2025.]*

## SUPPLEMENTAL MATERIAL

Supplemental material mbio.03369-24-s0001.docx Supplemental figures and table.

ASM does not own the copyrights to Supplemental Material that may be linked to, or accessed through, an article. The authors have granted ASM a non-exclusive, world-wide license to publish the Supplemental Material files. Please contact the corresponding author directly for reuse.
