# Erratum for Woo et al., “*Acinetobacter baumannii* OmpA hinders host autophagy via the CaMKK2-reliant AMPK-pathway”

**DOI:** 10.1128/mbio.01224-25

**Published:** 2025-05-08

**Authors:** Kyungho Woo, Dong Ho Kim, Ho-Sung Park, Man Hwan Oh, Je Chul Lee, Chul Hee Choi

## ERRATUM

Volume 16, no. 4, e03369-24, 2025, https://doi.org/10.1128/mbio.03369-24. Page 1, byline: Affiliation designation 3 (for Department of Medical Science, School of Medicine, Chungnam National University, Daejeon, South Korea) should be added for Kyungho Woo and Dong Ho Kim.

