# Supplementary material for: Acinetobacter baumannii OmpA hinders host autophagy via the CaMKK2-reliant AMPK-pathway
Source: mBio. 2025 Feb 25;16(4):e03369-24. doi: 10.1128/mbio.03369-24 (PMC11980379; doi:10.1128/mbio.03369-24)
Supplement: Supplemental material — Supplemental figures and table. [file mbio.03369-24-s0001.docx]

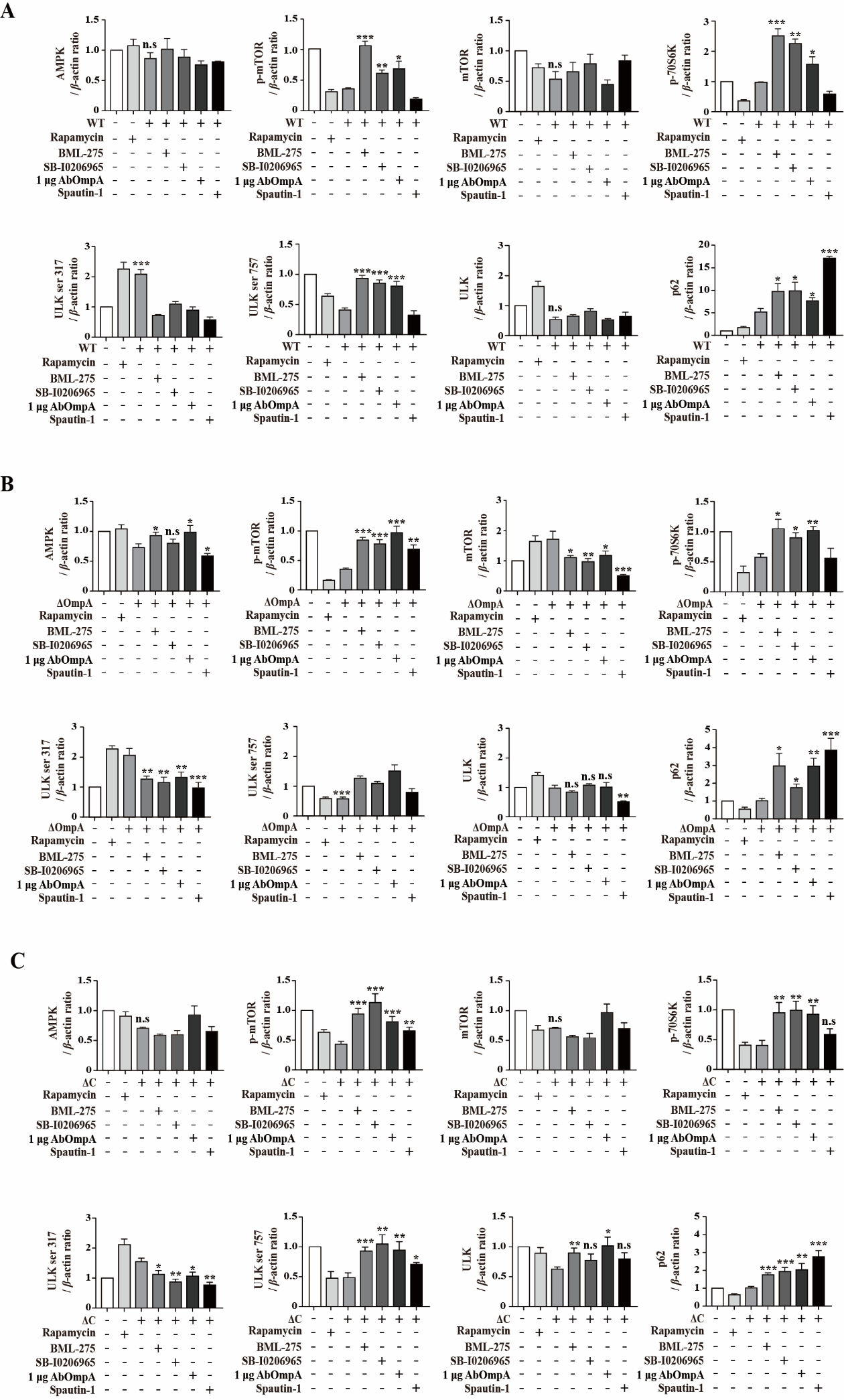


**Supplementary Figure 1.** Western blot results in Figure 3 were quantitated and presented in the graphs. All signal molecules were analyzed using Western blot: (A) Figure 3A, (B) Figure 3B and (C) Figure 3C. The levels of target proteins were normalized to those of *β*-actin and are presented as mean ± SEM of three independent experiments. ****p* < 0.001, ***p* < 0.01 and **p* < 0.05 *versus* each of infection condition.


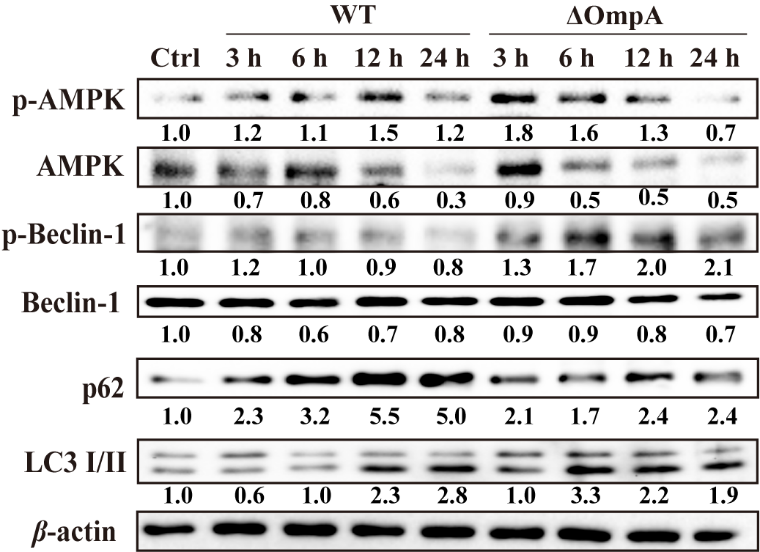


**Supplementary Figure 2.** *Acinetobacter baumannii* induces autophagy. RAW 264.7 cells were infected with *A. baumannii* ATCC 17978 wild-type (WT) and *ompA*-deletion mutant strains (ΔOmpA) at a multiplicity of infection (MOI) of 100 in a time-dependent manner. Target proteins levels were normalized to those of *β*-actin. Ctrl, negative control.


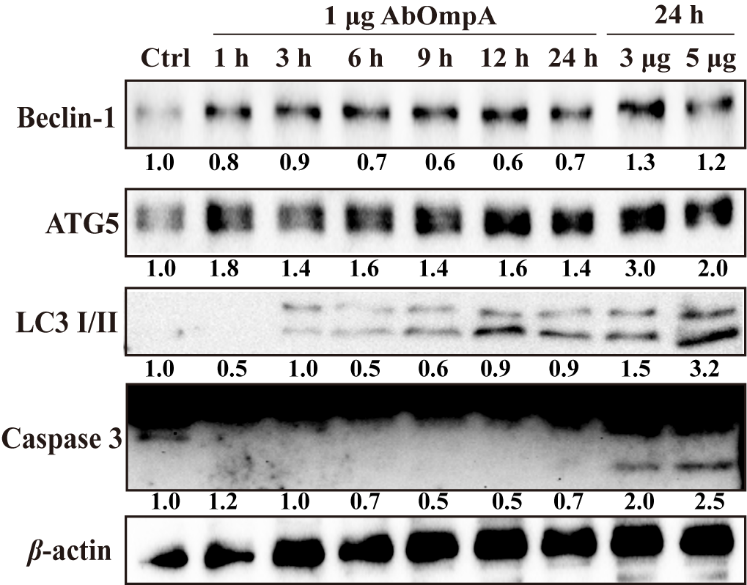


**Supplementary Figure 3.** AbOmpA induces autophagy and apoptosis. RAW 264.7 cells were treated with exogenous AbOmpA in a dose- and time-dependent manner. Target proteins levels were normalized to those of *β*-actin. Ctrl, negative control.

**
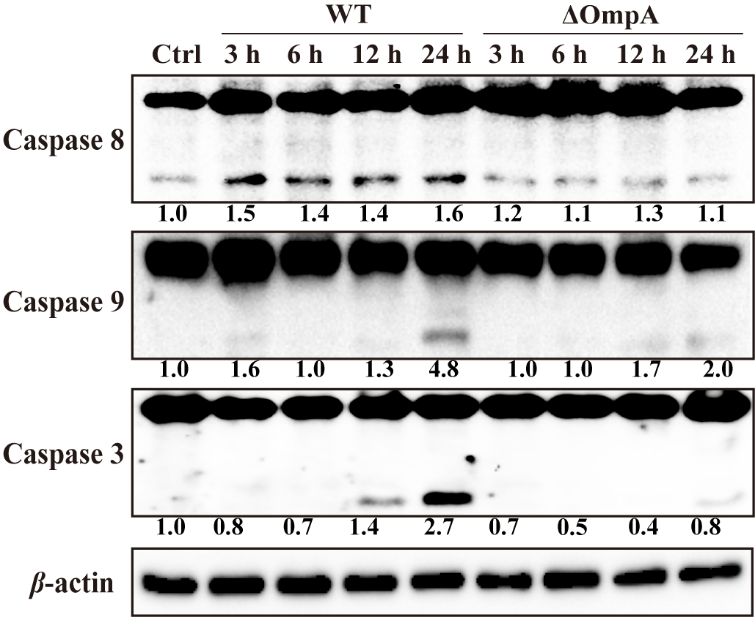
**

**Supplementary Figure 4.** *Acinetobacter baumannii* induces apoptosis. RAW 264.7 cells were infected with *A. baumannii* ATCC 17978 wild-type (WT) and *ompA*-deletion mutant strains (ΔOmpA) at a multiplicity of infection (MOI) of 100 in a time-dependent manner. Target protein levels were normalized to those of *β*-actin. Ctrl, negative control.


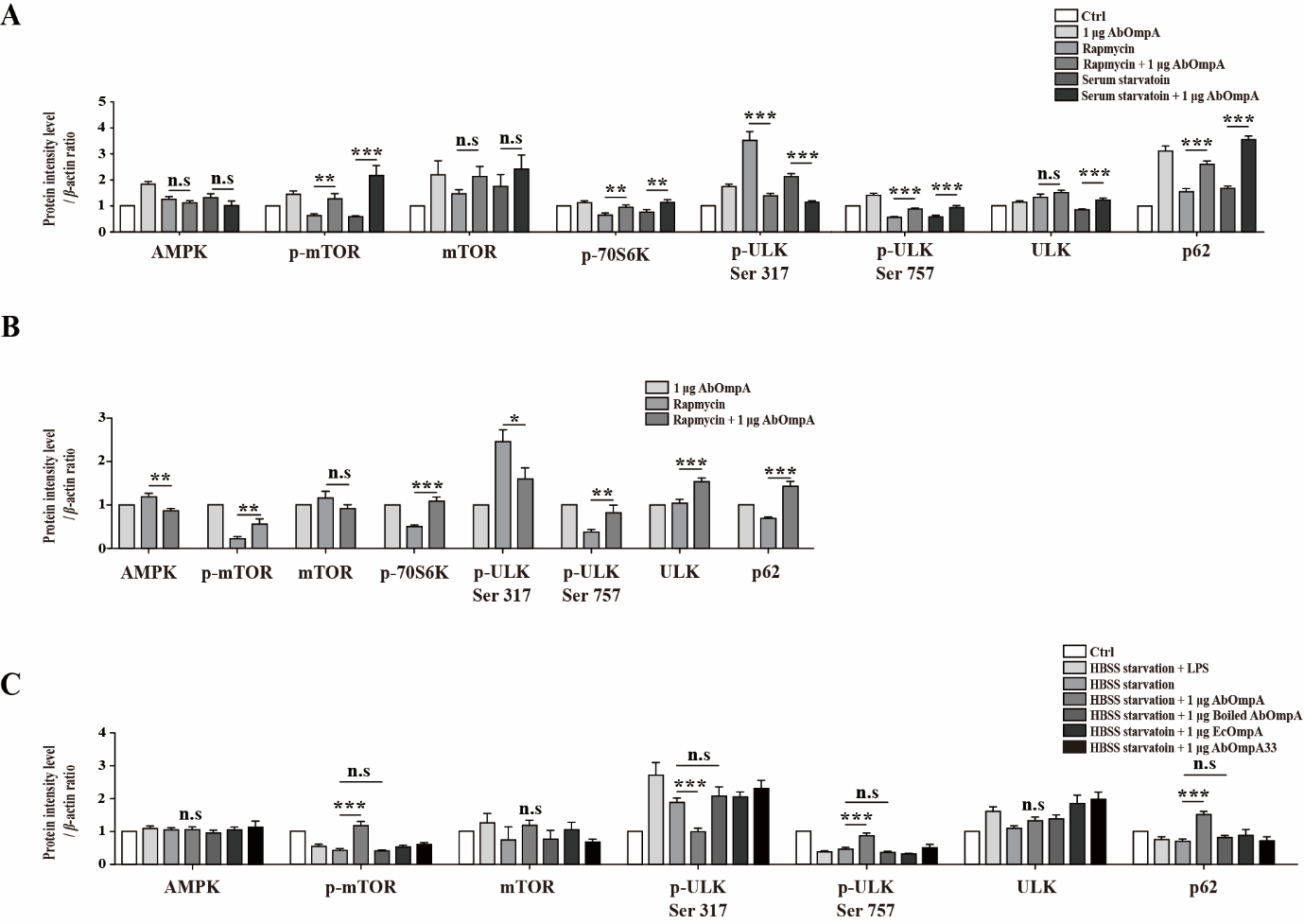


**Supplementary Figure 5.** Western blot results in Figure 5 were quantitated and presented in the graphs. All signal molecules were analyzed using Western blot: (A) Figure 5A, (B) Figure 5B and (C) Figure 5D. The levels of target proteins were normalized to those of *β*-actin and are presented as the mean ± SEM of three independent experiments. ****p* < 0.001, ***p* < 0.01 and **p* < 0.05 *versus* each of treated condition.


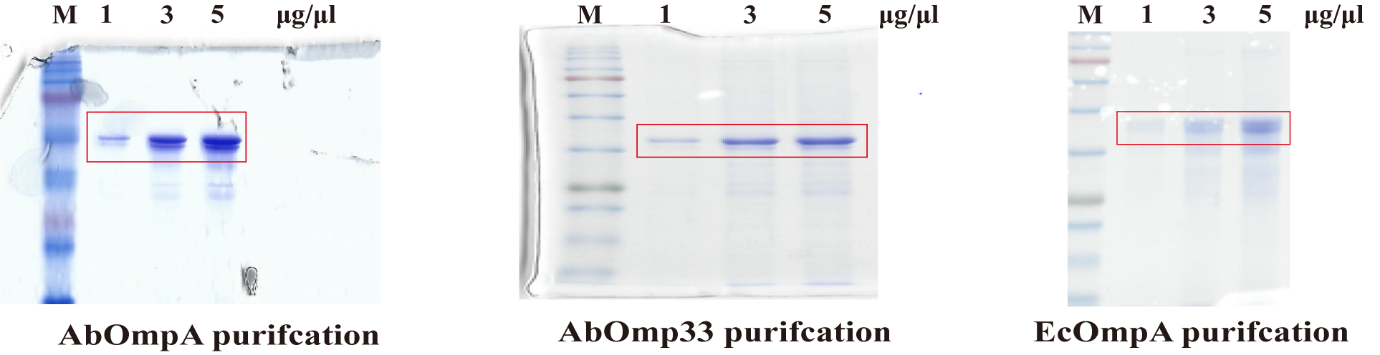


**Supplementary Figure 6.** Purification of outer membrane proteins. (**A**) AbOmpA, (**B**) AbOmp33, and (**C**) EcOmpA were purified using SDS-PAGE. Red square box indicates the targets band. M, size marker.


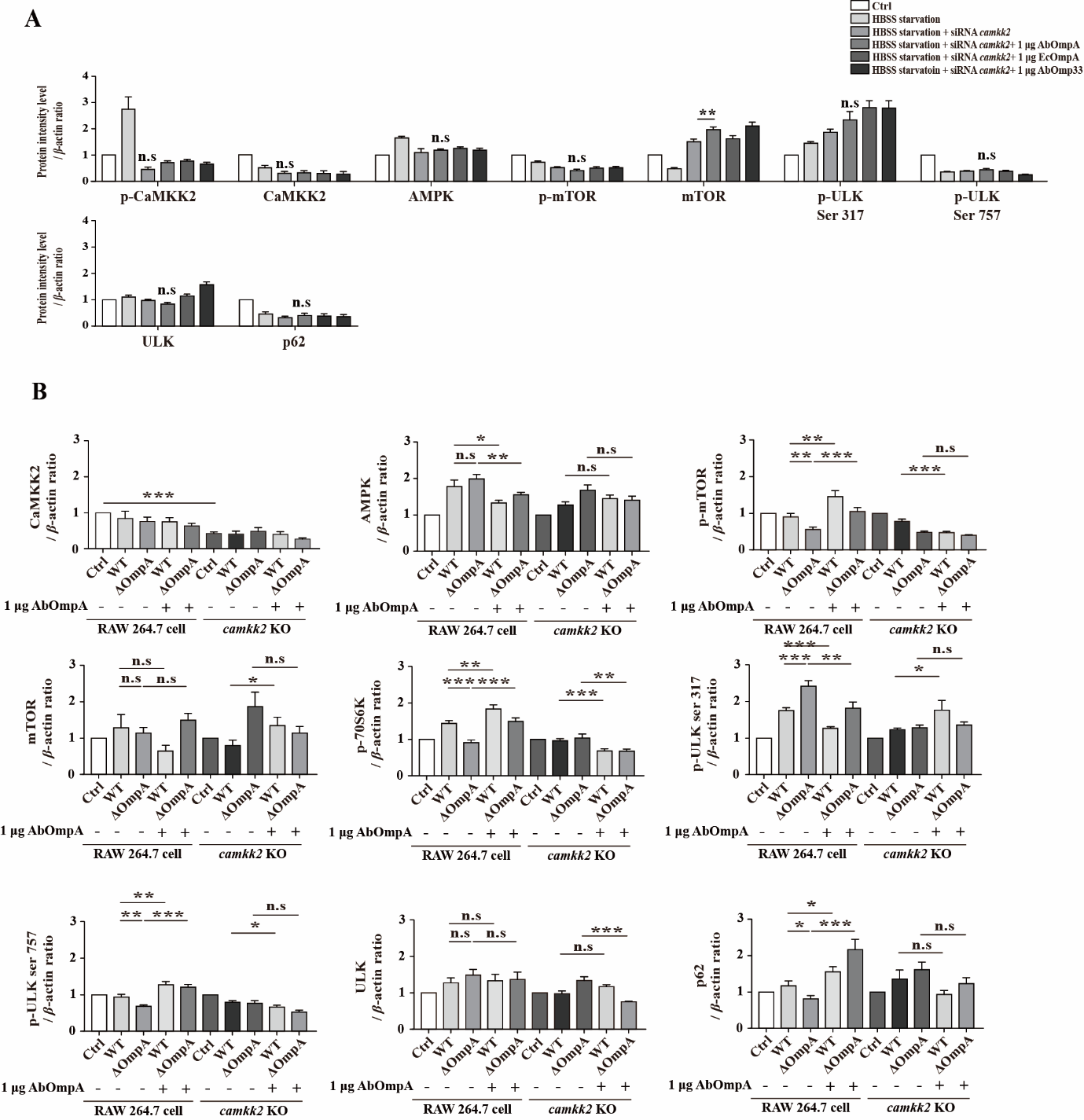


**Supplementary Figure 7.** Western blot results in Figure 6 were quantitated and presented in the graphs. All signal molecules were analyzed using western blot: (A) Figure 6C and (B) Figure 6E. The levels of target proteins were normalized to those of *β*-actin and are presented as the mean ± SEM of three independent experiments. ****p* < 0.001, ***p* < 0.01 and **p* < 0.05 *versus* each of treated condition.

#
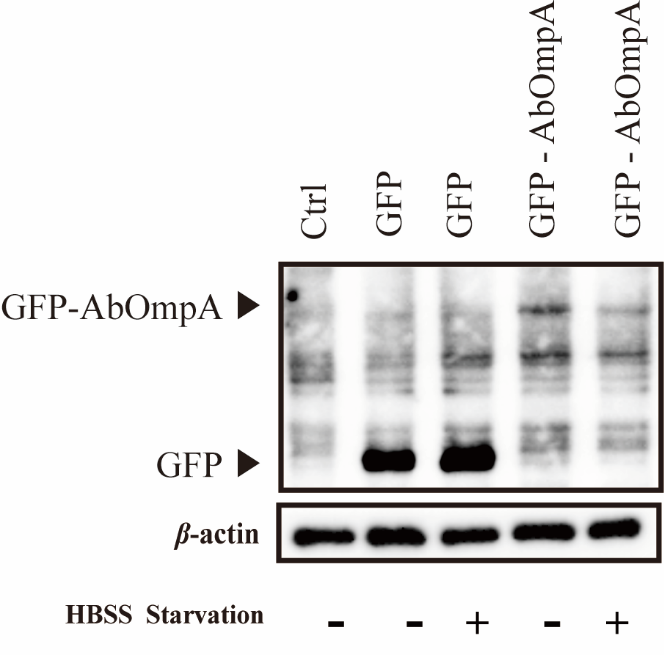


# Supplementary Figure 8. Co-IP analysis with GFP-AbOmpA. RAW 264.7 cells transfected with GFP empty vector and GFP-AbOmpA and then subjected to HBSS starvation for 1 h. Whole cell lysates (pull-down) were subjected to immunoblotting with GFP antibodies. Ctrl, negative control.


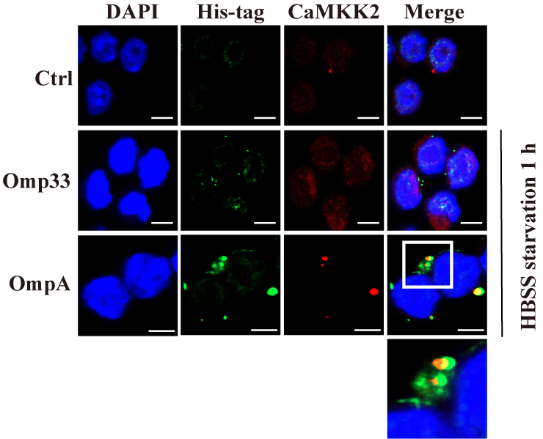


# Supplementary Figure 9. Colocalization of CaMKK2 and AbOmpA. RAW 264.7 cells subjected to HBSS starvation for 1 h. Cells were treated with exogenous AbOmpA (1 μg) and Omp33 (1 μg). CaMKK2 (red) and his-tag immunofluorescence (green) were monitored using confocal microscopy. Scale bar: 5 μm. Ctrl, negative control.


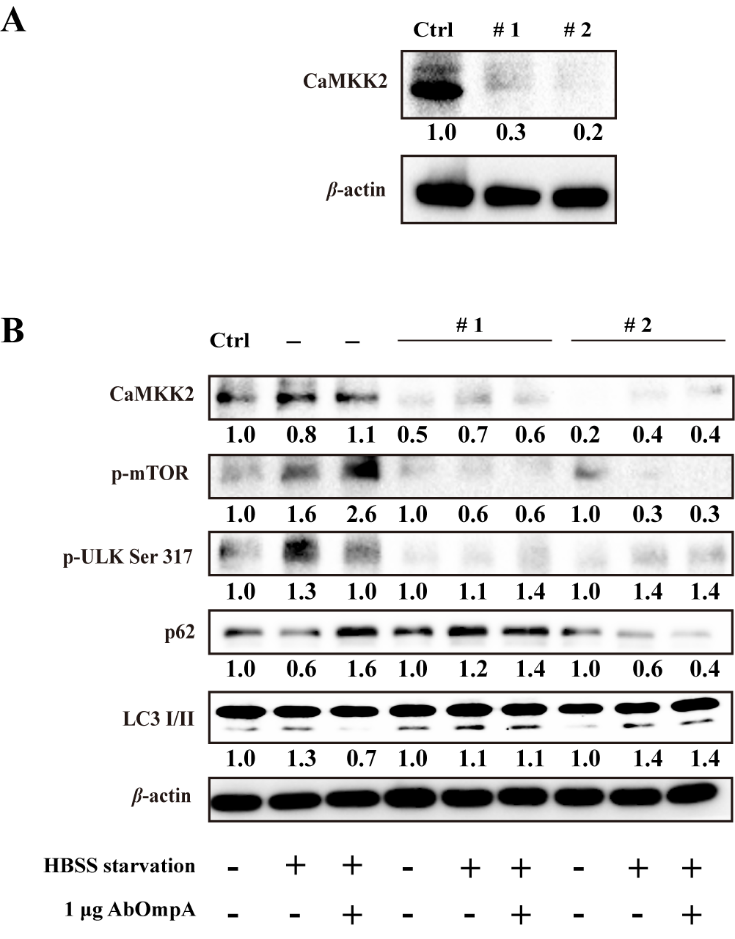


**Supplementary Figure 10.** Generation of *camkk2*-knockout cells using CRISPR/Cas9. (**A**) RAW 264.7 cells deficient of the *camkk2* gene were analyzed using western blotting. (**B**) RAW 264.7 cells and *camkk*2-knockout cells were subjected to HBSS starvation (Hank’s Balanced Salt Solution without calcium) for 1 h and then simultaneously treated with exogenous AbOmpA (1 μg). Target protein levels were normalized to those of *β*-actin. Ctrl, negative control.

**Table S1.**

**Gene** **Sequence (5′- 3′)**

| **Protein Purification** |  |  |  |
| --- | --- | --- | --- |
| *ompA* | *A. baumannii* | Fw | GGGGGGATCCGATGAAATTGAGTCGTATTGC |
|  |  | Rv | GGGGAAGCTTTTGAGCTGCTGCAGGAGCTG |
| *omp33* | *A. baumannii* | Fw | GGGGGGATCCGATGAAAAAACTTGGTTTAGCC |
|  |  | Rv | GGGGGAGCTCGCGAAACGGAATTTAGCATTTA |
| *ompA* | *E. coli* | Fw | GGGGGAATTCGATGAAAAAGACAGCTATCGC |
|  |  | Rv | GGGGAAGCTTAGCCTGCGGCTGAGTTACAA |
| **P-GFP -OmpA** | | | |
| *ompA* | *A. baumannii* | Fw | GGGGAAGCTTGCATGAAATTGAGTCGTATTGC |
|  |  | Rv | GGGGGGATCCTTGAGCTGCTGCAGGAGCTG |
| **P-GFP-LC3** | | | |
| *LC3* | *Mus musculus* | Fw | AAGCTTCGATGCCGTCCGAGAAGACCTT |
|  |  | Rv | GGATCCCACAGCCATTGCTGTCCCGA |
